# Treatment with *Anaerobutyricum soehngenii*: a pilot study of safety and dose–response effects on glucose metabolism in human subjects with metabolic syndrome

**DOI:** 10.1038/s41522-020-0127-0

**Published:** 2020-03-27

**Authors:** Pim W. Gilijamse, Annick V. Hartstra, Evgeni Levin, Koen Wortelboer, Mireille J. Serlie, Mariette T. Ackermans, Hilde Herrema, Aart J. Nederveen, Sultan Imangaliyev, Steven Aalvink, Morton Sommer, Han Levels, Erik S. G. Stroes, Albert K. Groen, Marleen Kemper, Willem M. de Vos, Max Nieuwdorp, Andrei Prodan

**Affiliations:** 1 Department of Vascular Medicine, Amsterdam University Medical Centers, Amsterdam, The Netherlands; 2 Department of Endocrinology and Metabolism, Amsterdam University Medical Centers, Amsterdam, The Netherlands; 3 Laboratory of Endocrinology, Amsterdam University Medical Centers, Amsterdam, The Netherlands; 4 Department of Radiology, Amsterdam University Medical Centers, Amsterdam, The Netherlands; 5 Laboratory of Microbiology, Wageningen University, Wageningen, The Netherlands; 6 DTU Biosustain, Copenhagen, Denmark; 7 Department of Clinical Pharmacy, Amsterdam University Medical Centers, Amsterdam, The Netherlands; 8 Human Microbiome Research Program, Faculty of Medicine, University of Helsinki, Helsinki, Finland

**Keywords:** Microbiome, Microbiota, Microbial ecology, Metagenomics

## Abstract

Dysbiosis of the intestinal microbiota has been implicated in insulin resistance, although evidence regarding causality in humans is scarce. We performed a phase I/II dose-finding and safety study on the effect of oral intake of the anaerobic butyrogenic strain *Anaerobutyricum soehngenii* on glucose metabolism in 24 subjects with metabolic syndrome. We found that treatment with *A. soehngenii* was safe and observed a significant correlation between the measured fecal abundance of administered *A. soehngenii* and improvement in peripheral insulin sensitivity after 4 weeks of treatment. This was accompanied by an altered microbiota composition and a change in bile acid metabolism. Finally, we show that metabolic response upon administration of *A. soehngenii* (defined as improved insulin sensitivity 4 weeks after *A. soehngenii* intake) is dependent on microbiota composition at baseline. These data in humans are promising, but additional studies are needed to reproduce our findings and to investigate long-term effects, as well as other modes of delivery.

## Introduction

The development of culture-independent approaches using high-throughput sequencing^1^ has drastically advanced knowledge of the gut microbiome, linking pathophysiology of metabolic diseases such as obesity and type 2 diabetes to an altered gut microbiota composition both in human and animal models^2,3^. However, the role of the intestinal microbiota and the mechanism mediating its impact on metabolic function in humans is still poorly understood. The technique of fecal microbiota transplantation (FMT) has provided insights as to the effects of gut microbiota on human metabolism^4^. In a pilot study, we showed that lean donor gut microbiota infusion was associated with an increase of relative abundance of *Anaerobutyricum* spp. in the (small) intestine, which was directly correlated with improvement in peripheral insulin sensitivity (Rd)^5^.

*Anaerobutyricum soehngenii* (previously designated *Eubacterium hallii strain* L2-7) is an anaerobic Gram-positive, catalase-negative bacterium belonging to the clostridial cluster XIVa of the phylum Firmicutes^6^. *A. soehngenii* has bile acid sodium symporter and choloylglycine hydrolase genes and is therefore capable of affecting host bile acid metabolism^7–9^. It is a butyrate-producing species, but in contrast to other well known human isolates such as *Roseburia* and *Faecalibacterium* spp. that produce butyrate from sugars, *A. soehngenii* has the capacity to produce butyrate from d- and l-lactate and acetate in an acid environment^8^, making it more likely to survive the passage through the gastrointestinal tract and the related exposure to low pH values. It is known that treatment-naive insulin resistant subjects have increased small intestinal levels of lactate-producing bacteria^10^, as well as increased intestinal lactate levels^11^. Moreover, human subjects with insulin resistance are characterized by increased production of lactate, which correlates with glucose metabolism^12^. *A. soehngenii* can convert a potentially damaging acid (e.g., lactic acid) into butyrate, a short-chain fatty acid (SCFA), which with known beneficial effects on glucose metabolism^13^, thus underscoring the potential therapeutic validity and mode of action of this strain. Previously, the safety and efficacy of oral *A. soehngenii* supplementation was shown in an animal model of insulin resistance by performing 4-week daily oral administrations in a dose-finding study in *db/db* mice^14^. Moreover, we observed a dose-dependent effect of *A. soehngenii* on improved insulin sensitivity in correspondence with fecal *A. soehngenii* levels. In these treated *db/db* mice we also observed beneficial effects on the expression of liver genes involved in lipolysis and steatosis, as well as changes in bile acids^14^.

While knowledge regarding the relationship between bacteria and metabolism in rodent models is rapidly increasing, confirmed causality of gut microbiota strains involved in human metabolism is still limited. Thus, in order to investigate the validity of murine data for human insulin sensitivity, we performed a single-blinded phase I/II dose-finding trial to determine the safety, efficacy, and optimal dosage of a live *A. soehngenii* strain orally ingested once daily for 4 weeks in treatment-naive males with metabolic syndrome. Our primary objective was to assess safety and to study the potential clinical impact on insulin sensitivity, as well as on lipolysis upon 4 weeks daily oral treatment with *A. soehngenii*. Changes in bile acid metabolism, MRI-measured liver fat content, and bowel habits were also studied. Finally, changes in intestinal microbiota composition, *Anaerobutyricum* spp. growth rates, and persistence of administered *A. soehngenii* up to 2 weeks after cessation of treatment were measured.

## Results

### Baseline characteristics

We included 27 overweight or obese Caucasian males with insulin resistance. Supplementary data sheet 1 shows that all included subjects had insulin resistance based on the presence of one of the three insulin resistance parameters (increased fasting glucose, increased fasting triglycerides, or increased homeostatic model assessment (HOMA)) either at screening or at the baseline study visit day. They were randomized to receive 10^7^ cells/day, 10^9^ cells/day, or 10^11^ cells/day of *A. soehngenii* each day for 4 weeks. During the trial, three subjects were excluded (one subject from each group) due to technical difficulties resulting in incomplete measurements and thus 24 were left for analyses. No significant differences were found in baseline characteristics between groups (Table 1).

**Table 1:** Baseline characteristics and safety parameters.

	All subjects	*n* = 24	*p*	10^5^ cells/ml	*n* = 8	*p*	10^7^ cells/ml	*n* = 8	*p*	10^9^ cells/ml	*n* = 8	*p*	p*	p**
	Week 0	Week 4		Week 0	Week 4		Week 0	Week 4		Week 0	Week 4			
Age, *years*	54 [46–69]			60 [52–68]			47 [43–60]			56 [43–59]			0.078	
Weight, *kg*	103 [92–111]	103 [91–111]	0.749	99 [87–110]	99 [87–108]	0.092	110 [99–117]	110 [99–118]	0.672	98 [87–109]	99 [88–110]	0.161	0.339	0.065
BMI, *kg/m*^*2*^	33 [30–33]	32 [30–33]	0.750	32 [29–34]	31 [29–34]	0.237	33 [31–33]	32 [31–33]	0.779	31 [29–34]	31 [29–34]	0.069	0.555	0.064
Waist circumference, *cm*	112 [107–118]	113 [106–117]	0.850	110 [105–114]	110 [105–115]	0.850	116 [109–118]	116 [111–117]	0.850	110 [104–120]	110 [104–120]	0.773	0.354	0.913
Fasting glucose, *mmol/l*	5.6 [5.2–5.8]	5.4 [5.2–5.6]	0.280	5.6 [5.2–5.9]	5.4 [4.9–5.8]	0.235	5.5 [5.1–5.9]	5.4 [5.2–5.9]	0.863	5.4 [5.2–5.9]	5.4 [5.1–5.6]	0.497	0.868	0.763
Insulin, *pmol/l*	85 [60–143]	97 [62–147]	0.668	86 [60–139]	79 [52–141]	0.484	98 [78–168]	126 [71–163]	0.327	79 [40–186]	102 [47–132]	0.292	0.907	0.309
HOMA-IR	3.1 [2.1–5.2]	3.4 [2.1–4.7]	0.689	3.3 [2.0–5.3]	2.4 [1.9–4.7]	0.401	3.4 [2.5–5.9]	4.2 [2.4–5.8]	0.093	2.9 [1.4–7.0]	3.4 [1.7–4.6]	0.233	0.912	0.172
Cholesterol														
Total, *mmol/l*	5.7 [4.8–6.4]	5.7 [4.7–6.4]	0.764	5.9 [4.1–6.4]	5.5 [4.2–6.7]	0.889	5.6 [4.5–6.1]	5.8 [4.3–6.3]	0.889	5.7 [4.9–7.2]	5.7 [4.9–6.4]	0.888	0.505	0.987
LDL, *mmol/l*	3.9 [3.0–4.2]	3.8 [2.8–4.2]	0.370	3.8 [2.7–4.1]	3.7 [2.5–4.6]	1.000	3.5 [2.8–4.2]	3.7 [2.6–4.1]	0.484	4.0 [3.4–5.0]	3.8 [3.3–4.4]	0.327	0.430	0.777
HDL, *mmol/l*	1.0 [0.9–1.2]	1.0 [0.9–1.1]	0.931	1.1 [0.8–1.2]	1.1 [0.8–1.2]	0.866	0.9 [0.9–1.1]	0.9 [0.9–1.1]	0.351	1.1 [0.9–1.3]	1.0 [0.9–1.2]	0.236	0.378	0.304
Triglycerides, *mmol/l*	1.7 [1.4–1.8]	1.8 [1.4–2.4]	0.493	1.6 [1.4–2.5]	1.7 [1.3–1.9]	0.575	1.7 [1.4–2.3]	2.2 [1.2–2.5]	0.779	1.6 [1.3–1.9]	1.9 [1.2–2.7]	0.161	0.854	0.658
Systolic blood pressure, *mmHg*	140 [130–148]	138 [131–144]	0.082	144 [127–161]	141 [129–159]	0.609	142 [130–156]	141 [135–150]	0.482	137 [124–144]	134 [114–139]	**0.028**	0.578	0.058
Diastolic blood pressure, *mmHg*	92 [86–95]	85 [80–90]	**0.002**	90 [79–99]	85 [79–93]	0.237	95 [87–98]	88 [74–94]	0.069	89 [85–94]	83 [76–89]	**0.012**	0.390	0.581
Creatinine, *µmol/l*	87 [79–93]	84 [78–91]	0.236	88 [81–93]	85 [78–94]	0.172	89 [73–96	85 [77–91]	0.482	85 [73–91]	83 [73–93]	0.752	0.578	0.452
Hemoglobin, *mmol/l*	9.1 [8.6–9.5]	8.9 [8.6–9.5]	0.519	9.0 [8.6–9.5]	8.9 [8.5–9.2]	0.621	9.0 [8.6–9.9]	9.2 [8.7–9.7]	0.246	9.3 [8.4–9.7]	8.9 [8.4–9.6]	**0.039**	0.850	0.179
Leukocytes, *10*^*e*^*9/l*	6.0 [5.3–7.1]	5.8 [5.4–6.9]	0.768	5.6 [4.5–6.0]	5.7 [4.6–5.9]	0.598	6.0 [5.2–6.9]	6.2 [5.3–6.6]	0.944	6.9 [4.8–8.4]	6.6 [4.7–8.2]	1.000	0.202	0.970
Thrombocytes,*10*^*e*^*9/l*	212 [194–252]	210 [206–241]	0.119	222 [186–254]	213 [185–245]	0.832	204 [196–242]	210 [202–237]	0.092	221 [190–300]	214 [207–267]	0.345	0.883	0.325
AST, *U/l*	25 [22–28]	27 [24–29]	0.182	24 [22–28]	26 [23–29]	0.291	27 [21–31]	26 [23–29]	0.619	25 [22–31]	28 [23–33]	0.105	0.741	0.305
ALT, *U/l*	33 [24–39]	30 [24–38]	0.626	34 [21–39]	29 [22–39]	0.798	36 [20–46]	32 [20–47]	0.916	31 [24–34]	30 [23–37]	0.611	0.873	0.964
AP, *U/l*	64 [55–77]	69 [57–78]	0.077	61 [50–82]	69 [51–79]	0.325	61 [50–75]	63 [50–74]	1.000	70 [60–90]	75 [60–89]	0.079	0.489	0.294
γGT, *U/l*	32 [22–43]	29 [22–42]	0.369	33 [20–42]	30 [20–42]	0.833	29 [21–43]	30 [22–40]	0.916	34 [24–68]	28 [23–63]	0.345	0.690	0.809
CRP, *mg/ml*	1.8 [1.0–4.8]	2.2 [1.0–4.1]	0.856	1.2 [0.8–3.9]	2.8 [1.1–5.1]	0.091	3.7 [0.8–9.5]	1.9 [1.1–3.4]	0.310	1.8 [1.0–5.1]	2.5 [0.9–4.4]	0.786	0.558	0.161
Caloric intake, *kcal/day*	1791 [1568–2028]	1871 [1694–1969]	0.798	1717 [1322–2002]	1676 [1519–1817]	0.893	1801 [1599–2028]	1785 [1594–2131]	0.753	1963 [1566–2424]	1969 [1890–2354]	0.401	0.305	0.739
Fat intake, *g*	75 [54–88]	65 [46–86]	0.449	60 [46–77]	48 [41–65]	0.109	76 [52–87]	55 [43–82]	0.345	83 [60–100]	86 [78–107]	0.093	0.297	**0.045**
Protein intake, *g*	84 [68–101]	92 [69–108]	0.717	80 [56–99]	92 [67–103]	0.345	98 [79–104]	92 [57–120]	0.917	74 [67–124]	90 [67–125]	0.889	0.641	0.777
Fiber intake, *g*	14 [13–19]	16 [12–22]	0.190	14 [14–19]	17 [14–24]	0.141	16 [13–20]	18 [9–26]	0.458	14 [12–23]	15 [10–24]	0.574	0.958	0.794
Carbohydrate intake, *g*	179 [165–200]	188 [173–250]	**0.036**	169 [158–195]	178 [164–217]	0.138	184 [157–196]	215 [184–263]	0.116	184 [167–215]	175 [167–278]	0.401	0.502	0.443
Compliance, *%*	100	100		100	100		100	100		100	100			
qPCR fecal *A. soehngenii*, gene copies/g feces	1.6 × 10^6^ [0.5 × 10^6^–3.2 × 10^6^]	1.2 × 10^9^ [2.6 × 10^7^–7.3 × 10^9^]	**1.2** × **10**^**−7**^	1.8 × 10^6^ [1.4 × 10^6^–3.7 × 10^6^]	1.4 × 10^7^ [5.4 × 10^6^–2.1 × 10^7^]	**0.012**	0.9 × 10^6^ [0.2 × 10^6^–2.3 × 10^6^]	1.7 × 10^9^ [8.6 × 10^8^–2.7 × 10^9^]	**0.012**	1.4 × 10^6^ [0.5 × 10^6^–3.4 × 10^6^]	1.9 × 10^10^ [0.9 × 10^10^–3.6 × 10^11^]	**0.012**	0.505	**0.0001**

Shown are baseline characteristics at week 0 and safety parameters at week 0 and week 4 expressed as median [inter-quartile range] for all three treatment groups. *p*-values represent within-group changes between week 0 and 4 (*p*, paired Wilcoxon tests), between-group differences at baseline (p*, Kruskal–Wallis tests) and between-groups comparison of changes between week 0 and week 4 (p**, Kruskal–Wallis tests). A *p*-value < 0.05 was considered significant. Significant *p*-values are shown in bold. Measurement units are shown in italics.

*BMI* body mass index, *HbA1c* glycated hemoglobin, *HDL* high-density lipoprotein, *HOMA-IR* homeostatic model assessment of insulin resistance, *LDL* low-density lipoprotein, *AST* aspartate transaminase, *ALT* alanine transaminase, *AP* alkaline phosphatase, *γGT* gamma-glutamyltransferase, *CRP* c-reactive protein.

### Safety parameters

*A. soehngenii* administration was well tolerated and no side effects or serious adverse events attributed to the intervention were observed (Supplementary Table 1). No difference was found in compliance between the 3 groups (Table 1). We observed no differences in either bowel habits or in daily energy and macronutrient intake during the study in any of the treatment groups. There were no changes in safety laboratory parameters such as hematology, kidney and liver parameters, and inflammatory and cholesterol markers, except for a clinically insignificant reduction of hemoglobin levels in the high-dose group. Vital signs such as blood pressure remained the same, except for a slight but statistically significant decrease in systolic and diastolic blood pressure in the high-dose group. Furthermore, there was no change in body weight, fasting glucose and insulin levels, or HOMA-IR in either of the treatment groups (Table 1).

### Levels of endogenous *Anaerobutyricum* spp. and of administered *A. soehngenii*

**Fig. 1:**
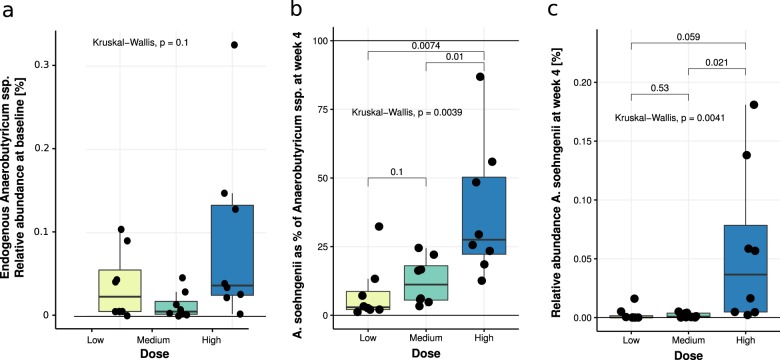
Proportion and abundance of endogenous *Anaerobutyricum* spp. and of administered *A. soehngenii* per dose group. **a** Relative abundance of endogenous Anaerobutyricum spp. at baseline in each of the three dose groups; **b** proportion of *A. soehngenii* as percentage of total *Anaerobutyricum* spp. (administered + endogenous strains) at week 4; and **c** relative abundance abundance of *A. soehngenii* at week 4. The box depicts the inter-quartile range (IQR), with the center line showing the median. The upper whisker extends the largest value no further than 1.5*IQR from inter-quartile range. The lower whisker extends to the smallest value at most 1.5*IQR below inter-quartile range.

**Fig. 2:**
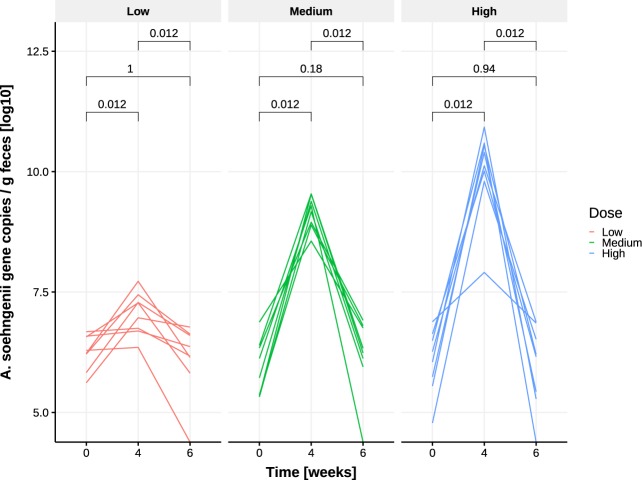
Presence of *A. soehngenii* as determined by qPCR at week 0 (baseline), upon 4 weeks of *A. soehngenii* dosing, and at week 6 (2 weeks after cessation of dosing), stratified per dose group. *p*-values are within-group comparisons (Wilcoxon-signed rank test). Values are Log-10 transformed.

Levels of endogenous *Anaerobutyricum* spp. were not significantly different in fecal baseline samples when comparing the three dose groups (Kruskal–Wallis, *p* = 0.10, Fig. 1a). Moreover, we found that both the proportion of *A. soehngenii* as of total fecal *Anaerobutyricum* spp. (Fig. 1b, *p* = 0.0039) and the relative abundance of *A. soehngenii* in the fecal microbiome (Fig. 1c, *p* = 0.0041) were significantly different among dose groups and were highest in the subjects who received the highest dose. Fecal *A. soehngenii* levels as determined by quantitative PCR (qPCR) were significantly increased after 4 weeks compared to baseline in all dosage groups (*p* = 0.012), with the highest increase in the high-dose group (Fig. 2 and Supplementary Table 2). After 2 weeks of cessation of dosing, qPCR showed that *A. soehngenii* was significantly reduced in each dose group to levels similar to those pre-treatment. Of note, there was a significant correlation between the qPCR-determined levels and the metagenomic-determined relative abundance of *A. soehngenii* (rho = +0.70, *p* = 0.0001). The estimated ratio of secreted/ingested *A. soehngenii* cells was found to be significantly higher in the low-dose group compared to the medium group (Wilcoxon *p* = 0.015) and the high-dose groups (Wilcoxon *p* = 0.00016, respectively) (Supplementary Fig. 1).

### Effect of *A. soehngenii* treatment on glucose metabolism and other metabolic parameters

**Fig. 3:**
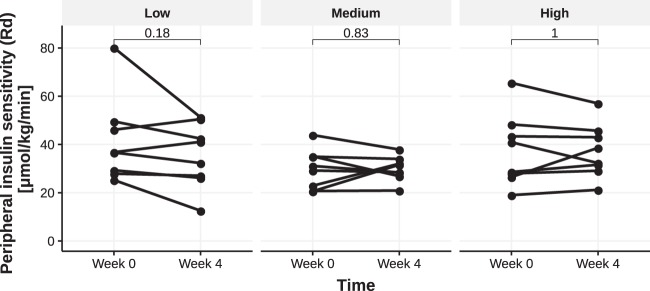
Peripheral insulin sensitivity Rd. Rd values before and after treatment with *A. soehngenii* in each dose group. *p*-values are within-group Wilcoxon-signed rank tests (paired).

**Fig. 4:**
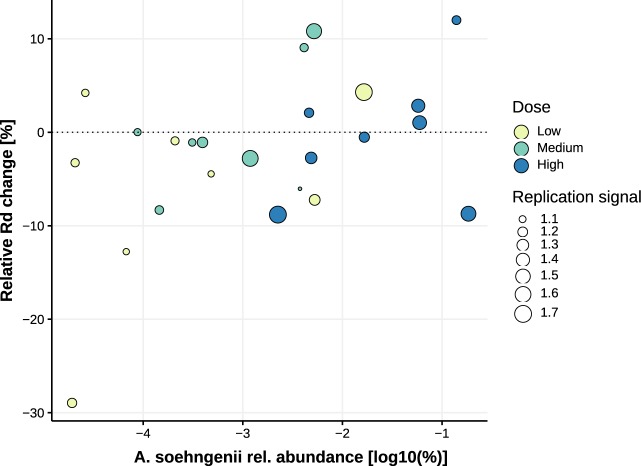
Scatter plot showing the correlation between the change in peripheral insulin sensitivity (Rd) and the relative abundance of administered *A. soehngenii* at week 4. Relative Rd change expressed as percentage change relative to baseline Rd values. Correlation assessed using Spearman’s rho (rho = +0.40, *p* = 0.05). Color shows dose groups; *Anaerobutyricum* spp. replication signal is represented by the area of the dots.

Insulin sensitivity was determined by performing hyperinsulinemic euglycemic clamps before and after treatment. We assessed insulin-mediated suppression of endogenous glucose production (EGP, a marker of hepatic insulin sensitivity) during the first step of the clamp and whole-body glucose rate of disposal (Rd) during the second step (Supplementary Table 3). We found no overall effect of *A. soehngenii* administration on either hepatic insulin sensitivity (EGP suppression) or Rd in either of the dose groups (Supplementary Table 4 and Fig. 3). Other metabolic parameters such as resting energy expenditure, insulin-mediated lipolysis determined as suppression of glycerol rate of appearance (Ra, a measure of adipose tissue insulin sensitivity), and free fatty acids (FFA) suppression (suppression of circulating plasma FFAs relative to basal state) were also not affected (Supplementary Table 4). However, when all treatment groups were pooled, the fecal relative abundance of administered *A. soehngenii* correlated positively and significantly with Rd (rho = 0.41, *p* = 0.044). There was a trend for a positive correlation between relative abundance of administered *A. soehngenii* and delta Rd, as well as the relative change in Rd (rho = 0.39, *p* = 0.061, and rho=0.40, *p* = 0.05, respectively) (Supplementary Table 5 and Fig. 4). We found no change in intrahepatic triglyceride (IHTG) content between or within treatment groups before or after intervention (Supplementary Fig. 2).

### Changes in SCFAs and bile acid metabolism before and after *A. soehngenii* treatment

**Fig. 5:**
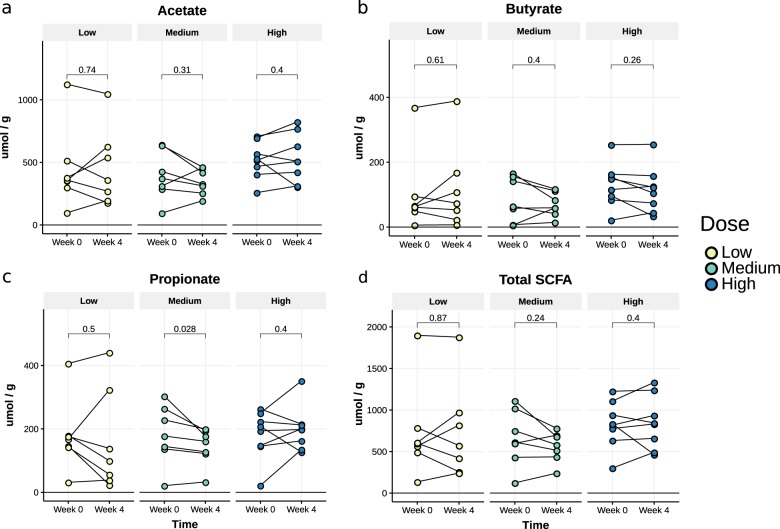
Fecal SCFA levels. Acetate (**a**), butyrate (**b**), propionate (**c**), and total (**d**) before and after *A. soehngenii* treatment in all dosage groups. Data are expressed as median [inter-quartile range]. *p*-values represent within-group changes between week 0 and week 4 (Wilcoxon tests). SCFA short-chain fatty acid.

**Fig. 6:**
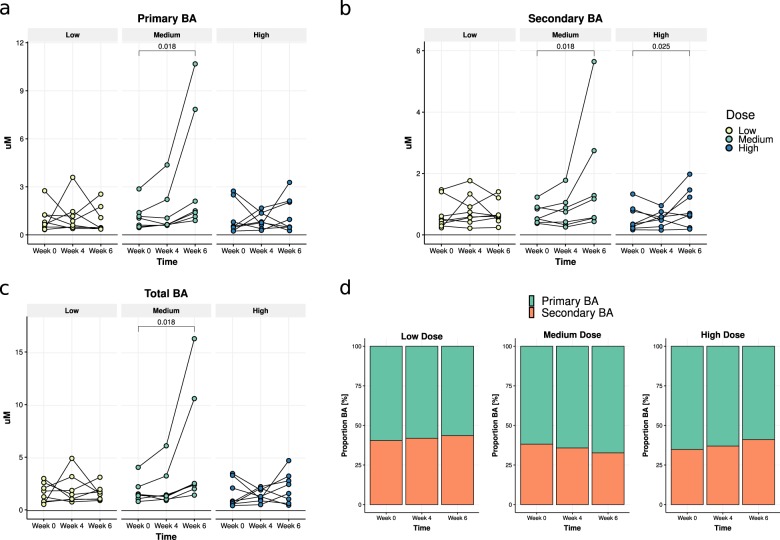
Plasma bile acids (BA). Change in plasma primary (**a**), secondary (**b**), and total bile acids (**c**) after 4 and 6 weeks; **d** the proportion of plasma primary and secondary bile acids, per dose group and time-point. Data are expressed as median [range]. *p*-values represent within-group differences between week 0 and week 6 (paired Wilcoxon-signed rank tests). BA bile acids.

We observed no differences in fecal SCFA levels before and after 4 weeks of treatment in the low and high-dose groups of daily oral *A. soehngenii* treatment. However, we observed a significant reduction in fecal propionate levels in the middle dose group (*p* = 0.028), from a median of 178 umol/g to 161 umol/g (Fig. 5c and Supplementary Table 6). In the same middle dose group, in line with our findings in insulin resistant mice^14^, we also observed a change in fasting plasma bile acids at 6 weeks, mainly due to an increase in secondary bile acids from a median level of 0.53 µM to 1.17 µM (*p* = 0.018) (Fig. 6 and Supplementary Table 7).

### Fecal microbial alpha- and beta-diversity in relation to metabolic response

Shotgun metagenomic sequencing was performed on DNA from fecal samples taken at baseline and after 4 weeks of *A. soehngenii* intervention. There were no significant changes in gut microbiota richness or in microbial alpha-diversity (Shannon index) among the different study groups, nor any significant between-group differences 4 weeks after treatment (Supplementary Fig. 3). There were no significant links between fecal microbial beta-diversity (assessed by Bray-Curtis dissimiliarity based on microbial composition) and response (Supplementary Fig. 4, PERMANOVA *p* > 0.05). Microbial composition at baseline and after 4 weeks of *A. soehngenii* administration is shown in Supplementary Fig. 5A, stratified by dose group.

### Replication activity of *Anaerobutyricum* spp

**Fig. 7:**
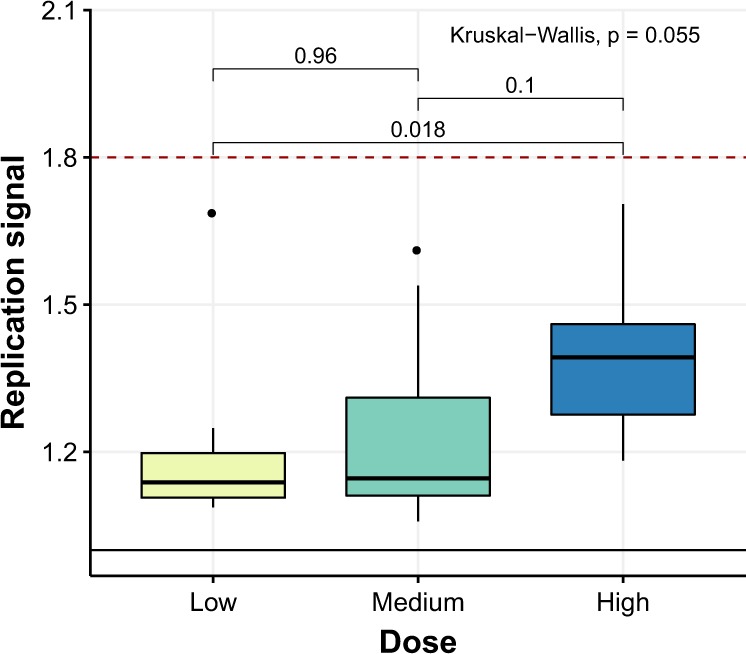
Replication activity of *Anaerobutyricum* spp. after 4 weeks of treatment per dose group. Data expressed as median and [inter-quartile range]. Within-group comparisons performed with Mann–Whitney *U-*tests; between-group comparison performed using a Kruskal–Wallis test. The box depicts the inter-quartile range (IQR), with the center line showing the median. The upper whisker extends the largest value no further than 1.5*IQR from inter-quartile range. The lower whisker extends to the smallest value at most 1.5*IQR below inter-quartile range.

The *A. soehngenii* present in the vials (measured in the high-dose samples) had a strong replication signal of 1.8, showing that ~80% of the cells in this sample were undergoing DNA replication, in line with the most probable number (MPN) culture-based assays showing high viability. Replication signal could only be determined jointly for all *Anaerobutyricum* spp. (endogenous *A. hallii* and administered *A. soehngenii* could not be distinguished) and was variable but consistently lower than in the administered drink. This indicates that *Anaerobutyricum* spp. in the fecal samples did not have a replication activity as high as that of the administered *A. soehngenii*. Post-treatment specimens did not exhibit a significantly different replication signal compared to pre-treatment samples (*p* = 0.74). However, the high-dose post-treatment samples containing over 25% *A. soehngenii* (Fig. 1b) showed higher replication compared to corresponding high-dose pre-treatment samples (*p* = 0.039). There was a borderline significant difference in replication signal between the different dose groups after treatment (Kruskal–Wallis, *p* = 0.055), with the high-dose group showing a significantly higher *Anaerobutyricum* spp. replication signal compared to the low-dose group (*p* = 0.018; Fig. 7).

### Responders versus non-responders analyses

We next performed an exploratory post hoc analysis to study which microbiota composition characteristics would differentiate metabolic responders from non-responders upon *A. soehngenii* treatment. We set a threshold of 4 µmol/kg/min as the minimum significant change in Rd. In effect, subjects whose Rd increased by at least 4 µmol/kg/min were classified as showing an “Increase”; subjects whose Rd decreased by at least 4 µmol/kg/min were classified as showing a “Decrease”; and subjects whose Rd changed by <4 µmol/kg/min (either increasing or decreasing) were labeled as showing “No change”. By this classification, five of the subjects showed an increase in Rd upon *A. soehgenii* administration (i.e., were responders), eight subjects showed a decrease, and 11 subjects showed no change. Microbial composition at baseline and after 4 weeks of *A. soehngenii* administration stratified by these Rd response groups is shown in Supplementary Fig. 5B. We next set out to identify baseline characteristics that discriminated subjects that showed an increase in Rd (from 26.5 µmol/kg/min at baseline to 38.6 µmol/kg/min at week 4) from subjects showing either a decrease or no change in Rd. Subsequent power calculations using the responders Rd data showed that 20 metabolic syndrome subjects per arm are needed to find a significant difference in Rd upon highest dose of *A. soehngenii*, when taking baseline fecal microbiota composition into account. The abundance of three intestinal microbial species in baseline fecal samples significantly correlated with clinical response (i.e., with change in Rd from baseline to week 4): *Eubacterium rectale* and *Lachnospiraceae* spp. showed a direct correlation (rho = +0.42, *p* = 0.041, and rho = +0.46, *p* = 0.024, respectively), whereas *Prevotella copri* showed an inverse correlation (rho = −0.41, *p* = 0.043). Interestingly, subjects showing a significant increase in Rd had around 3 times lower median baseline abundances of *P. copri* and *Ruminococcaceae* spp. (Supplementary Table 9). There was no difference between responders and non-responders with regard to daily caloric intake either at baseline (Wilcoxon *p* = 1.00) or after 4 weeks of *A. soehngeni* administration (Wilcoxon *p* = 0.75). There were no significant differences between responders and non-responders in total, conjugated, or unconjugated bile acids at either baseline or at week 4 (all Wilcoxon *p* > 0.05).

## Discussion

In this phase I/II single (only participant) blinded pilot trial, we tested safety and efficacy of *A. soehngenii* in metabolic syndrome subjects and found that daily ingestion of increasing *A. soehngenii* doses for 1 month was associated with increased fecal levels of *A. soehngenii* with greatest efficacy in the subjects who received the highest dose. The increase was transient (Supplementary Table 1 and Fig. 2), as 2 weeks after cessation most of the *A. soehngenii* was cleared from the feces. In line with our murine data^14^, we observed beneficial changes in bile acid metabolism, which combined with *Anaerobutyricum* spp. growth dynamics suggests that this bacterial strain survives passage through the gastrointestinal tract. Furthermore, we found that the relative abundance of administered *A. soehngenii* positively correlated with improved Rd (*p* = 0.044). Combined with the good safety profile, our data imply that the highest dose of the *A. soehngenii* is well tolerated and may be an additional treatment for insulin resistance.

This study takes the reductionist approach of reintroducing a bacterial therapeutic strain in metabolic syndrome subjects based on previous intervention trials^5^. We show that this approach is feasible, safe, and may induce a beneficial cardiovascular profile based on the introduced metabolically active bacterial strain. Interestingly, a recent paper showed that the increase in the levels of fecal propionate was causally linked to insulin resistance^15^, in line with our finding of decreased propionate upon *A. soehngenii* administration. However, both fecal and plasma SCFAs are notoriously difficult to measure due to volatility and assay detection limits^16^. Thus, the reduction in blood pressure in the highest dose group might be driven by SCFA-producing fecal bacterial strains^17^, despite not finding a significant effect on fecal SCFA levels. Finally, although the treatment efficacy of single-strain *A. soehngenii* was smaller than our findings on improved Rd upon lean donor FMT^5,10^, a recent FMT study from another group underscored these findings and showed that an enrichment of *Anaerobutyricum* spp. was associated with altered bile acids and clinical efficacy upon donor FMT in patients with ulcerative colitis^18^. In line, our results are similar to other human single-strain intervention studies demonstrating (in a subset of patients) an effect on insulin sensitivity after 12 weeks of supplementation with a high dose of *Lactobacillus reuteri* DSM 17938^19^. Upon the highest dose of *A. soehngenii*, half of metabolic syndrome subjects showed a significant improvement in glucose metabolism, paralleled by a concomitant increase in fecal levels of *A. soehngenii* after 4 weeks. This was corroborated by recent studies^20,21^ showing that engraftment of administered bacterial strains is only seen in a subset of treated subjects and depends on baseline fecal microbiota composition allowing engraftment and driving cohabitation between the endogenous microbiota and the exogenous bacterial strains^21^. We thus calculated that if baseline microbiota composition indeed drives the efficacy of engraftment of the *A. soehngenii*, we would need to treat about 20 metabolic syndrome subjects with this specific baseline microbiota composition with a dose of 10^11^ cells/day to be able to detect a significant increase in Rd. In contrast to our murine study^14^ and although fecal *A. soehngenii* increased significantly after 4 weeks of treatment, we observed no changes in fecal butyrate. While we cannot rule out that the effect of *A. soehngenii* administration is due to butyrate production^16^ from lactate and acetate in the small intestine^6,7^, we also observed that, upon *A. soehngenii* administration, plasma bile acid concentrations in the medium dose group changed with a predominant increase in plasma secondary bile acids, known to associate with improved glucose metabolism in insulin resistant subjects^22^. It has been previously observed in a human intervention trial using *B. infantis* that high concentrations (10^10^ CFU) of bacterial strains can induce a crowding effect resulting in less efficient dispersion of the bacteria in the intestine and thus in different clinical effects^23^. The fact that the medium group showed the most pronounced changes in bile acid composition may signify that it is the dose that best drives the endogenous-exogenous bacterial strain intestinal milieu for generation of secondary bile acids. Moreover, the reduction in fecal propionate levels in the same medium dose group, but not in other dose groups, aligns with this finding, although it may have been caused by the small number of subjects per treatment group. It has been increasingly recognized that intestinal microbiota play an important role in bile acid metabolism by synthesizing secondary bile acids from primary bile acids via deconjugation and dihydroxylation^24^. Next to their role in intestinal fat absorption, bile acids are crucial regulators of glucose and energy homeostasis^24^, and recent studies have shown that disturbances in bile acid metabolism may contribute to the pathogenesis of type 2 diabetes^1,25,26^. In line, our data in insulin resistant mice demonstrated that oral *A. soehngenii* treatment significantly changed levels of plasma bile acid^14^ and a previous probiotic human trial likewise showed altered plasma bile acids upon use of *L. reuteri* DSM 17938^19^.

Interestingly, the estimated number of *A. soehngenii* cells present in the fecal sample after 4 weeks of *A. soehngenii* administration was orders of magnitude higher than the daily intake of *A. soehngenii* cells per day (Supplementary Fig. 1). The ratio was significantly larger for the low dose compared to the high-dose group, suggesting that low amounts of *A. soehngenii* cells may be better protected by the milk drink during gastrointestinal passage. Another, non-exclusive, possibility is that there is a strong competition for resources in the colon and that low amounts of *A. soehngenii* can compete better and multiply more than higher dosages. The higher replication signal of *A. soehngenii* in the drink (freshly frozen and stored after being grown in single-culture condition on optimized culture media) compared to that of *Anaerobutyricum* spp. in feces likely reflects lower growth rates of *Anaerobutyricum* spp. (including *A. soehngenii)* in the limited substrate and high competitive environment of the gut. *A. soehngenii* was grown in large scale production in a sucrose-based medium, found in a previous study to be protective during frozen storage^27^.

The genome of *A. soehngenii* was recently published and underlined significant differences compared to the (endogenous) *A. hallii*^6^. Altogether with the different SCFA production pattern and bacterial wall fatty acid membrane composition of *A. soehngenii*, and in line with our clinical findings, these data strongly suggest that *A. soehngenii* has specific properties. Nevertheless, the dose-dependent increase in fecal *A. soehngenii* levels upon treatment was not associated with major changes in gut microbiota diversity, consistent with the observations from our mice study^14^. However, we observed an inverse correlation between baseline abundance of *P. copri* and the change in Rd (rho = −0.41, *p* = 0.043). Also, a comparison of responders and non-responders at baseline found that responders had around 65% less *P. copri* when compared to non-responders (Supplementary Table 9). The relation between *A. soehngenii* and *Prevotella copri* might be of interest, as the latter strain has been linked to glucose metabolism in humans and may work synergistically with *A. soehngenii* on insulin-sensitizing effects^28^. Thus, future studies will have to focus on dissecting the therapeutic synergy of co-administrating other bacterial strains, together with *A. soehngenii*.

The rapid decrease in fecal *A. soehngenii* levels after 2 weeks cessation of daily administration (Fig. 2) occurred at the same time as the increase in plasma primary bile acids (Fig. 6). This is similar to findings in the study with *L. reuteri*^19^, suggesting that systemic effects may persist for several weeks after the administered strain’s concentration in feces falls. As expected, beneficial metabolic effects were not seen in all subjects in the highest dosage groups. It is likely that the administered *A. soehngenii* is either not maximally engrafting or is not active enough to induce these effects. Another study in infants showed that the administered strain did not engraft in all treated subjects^29^, although the baseline microbiota composition was not considered.

Our study has several limitations, including the nature of its study design, single-blinded for the participant only. Although not as powerful as a randomized clinical trial (RCT) in determining treatment effect, a single-blinded, dose-escalation study was chosen instead due to ethical considerations, as an important aim of this study was to determine whether a high daily dose of 10^11 ^cells/day of *A. soehngenii* was safe and well tolerated in humans. During the trial, the viability of the 10 ml tubes that were stored at −80C was checked every 6 months. However, we did not determine the viability after home freezer storage during the 4 weeks intervention; we assumed viability loss, if any, to be similar in all households. The parameters used in the calculation of the ingested/secreted ratio of *A. soehngenii* may vary widely between as well as among individuals, thus the estimated ingested/secreted ratio values are approximations. Another limitation is the small group size and relatively short duration of treatment. When pooling subjects and looking at relative changes after 4 weeks of treatment, we observed a significant correlation between the relative abundance of administered *A. soehngenii* and the change in Rd. Thus, these outcomes could serve to guide power calculations for future intervention RCT trials with high-dosed bacterial strains such as *A. soehngenii*^30^. Moreover, as the goal of our study was to test safety and efficacy of different *A. soehngenii* dosages in humans, we did not compare different *A. soehngenii* strains, which will need to be done in future studies. The effect of the *A. soehngenii* on the phenotype of the participants may be mediated by unknown factors other than SCFA and secondary bile acids. Finally, stomach acid and oxygen affect viability of administered strains, which thus are independent of original ingested dose. However, the fact that the *A. soehngenii* strain showed the highest replication signal in the feces of subjects treated with the highest dose suggests that large daily amounts are needed. Future research will have to show whether protecting *A. soehngenii* against stomach acid and oxygen (e.g., by encapsulation and/or freeze-drying) will have greater therapeutic efficacy.

In conclusion, in this proof-of-concept pilot study, humans with metabolic syndrome were treated with a bacterial strain selected based on microbiota findings from our previous studies^5,10,14^. When all treatment groups were pooled, we observed a positive correlation between fecal *A. soehngenii* abundance and Rd. These results suggest that modulating the microbiota in humans may improve glucose metabolism and could therefore constitute a therapeutic modality in the treatment of type 2 diabetes. More research is needed on long-term effects and modes of delivery, which were beyond the scope of the current study. Nevertheless, we show here that using the current administration, high-dosed *A. soehngenii* is partially able to survive gastrointestinal tract passage and is accompanied by a beneficial safety profile. This provides a rationale for future *A. soehngenii* high-dose intervention trials in treatment-naive human subjects with metabolic syndrome.

## Methods

### Study subjects

Caucasian male subjects (*n* = 27) aged 21–69 years with metabolic syndrome^31^ not on any medication with a body mass index (BMI) between 25 and 43 kg/m^2^ and waist circumference > 102 cm, as well as either increased fasting plasma glucose (FPG) ≥ 5.6 mmol/l or increased fasting triglycerides ≥ 1.7 mmol/l, were recruited via local advertisements. Also, HOMA was calculated as an extra screening marker of insulin resistance (HOMA > 2.5). Exclusion criteria were the use of any medication, such as the use of proton pomp inhibitors (PPIs), statins, antihypertensives, oral anticoagulants, and antibiotics in the last 3 months, substance abuse (nicotine or drugs, alcohol > 2 units/day), and history of cholecystectomy or any chronic disorder with the exception of common obesity-related conditions. Only males were included as changes in female hormone concentrations in (postmenopausal) women have a disturbing effect on the insulin sensitivity^32^. Study participants were requested not to alter their physical exercise patterns after inclusion. All participants provided written informed consent and all study procedures were approved by the IRB (ethics committee) of the Amsterdam University Medical Center and conducted in accordance with the Declaration of Helsinki. The study was prospectively registered at the Dutch Trial registry (NTR4913, date of registration: 2014-11-22, https://www.trialregister.nl/trial/4775).

### Culturing of *A. soehngenii*

The cells were obtained by culturing *A. soehngenii* at 500-liter scale in a basic phosphate-bicarbonate salt medium containing 2% yeast extract, 0.4% soy peptone, and 2% sucrose, at pH 6.8 and 37 °C. Following autoclaving, filter-sterilized components were added, including cysteine (final concentration 0.05%) and a 1 ml per liter of a vitamin solution (containing per liter 10 mg biotin, 10 mg cobalamin, 30 mg para-aminobenzoic acid, 50 mg folic acid, and 150 mg pyridoxamine). A. *soehngenii* cells were harvested by microfiltration, washed with phosphate-buffered saline (PBS), and finally stored in PBS containing 10% glycerol at concentrations of either 10^6^ cells/ml (low dose), 10^8^ cells/ml (middle dose), or 10^10^ cells/ml (high dose) in 10 ml tubes at −80° C. *A. soehngenii* was handled under strict anaerobic conditions, which were maintained during all stages of the production of the concentrated cells: during growth, microfiltration, glycerol mixing, and filling of the tubes with a nitrogen atmosphere. The viability of *A. soehngenii* in randomly selected high-dose tubes (stored at −80° C at the AMC Department of Clinical Pharmacy) was tested every 6 months using MPN analysis in YCFA medium. MPN analyses were performed in duplicate in anoxic YCFA medium containing sucrose incubated at 37° C for 5 days. For the lowest dilutions three tubes were used, ranging up to 10^−11^. Growth was scored by visual and microscopic inspection. High-dose tubes stayed constant at 10^10^ cells/ml, within the errors of the MPN method. The drink containing *A. soehngenii* was shotgun sequenced using the same protocol as the study subjects’ fecal samples.

### Study design

**Fig. 8:**
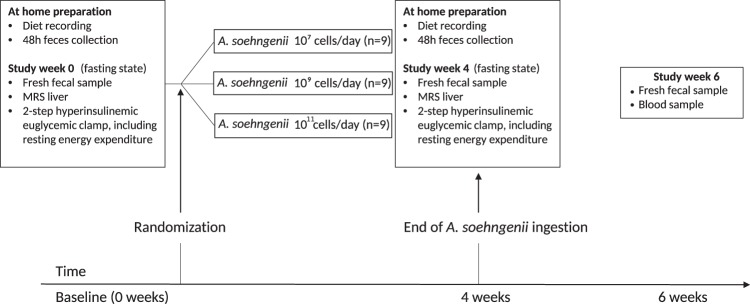
Study design. Diagram describing the study groups and showing the time-points of biological samplings and clinical measurements.

The study was set up as a phase I/II single center single (only participant) blinded dose-escalation trial in order to study treatment efficacy of each dose for future clinical trials. Subjects were assigned to 1 of 3 treatment arms (Fig. 8) where they consumed once-daily for 4 weeks, 10 ml of *A. soehngenii* strain L2-7 (NCBI taxonomy id 105843)^6^ at a concentration of either 10^6^ cells/ml (low dose), 10^8^ cells/ml (middle dose), or 10^10^ cells/ml (high dose). Thus, each subject received 10^7^ cells/day (low dose), 10^9^ cells/day (middle dose), or 10^11^ cells/day (high dose) once-daily for the duration of the 4 weeks administration period. Study subjects received the tubes containing the live *A. soehngenii* cells in frozen form and stored these in their home freezer at −20° C. Prior to use, a single 10 ml tube was thawed, the contents were mixed with 100 ml of milk and consumed fully. These concentrations are comparable to other human probiotics trials^19^. At every study contact, subjects visited the clinical research unit after an overnight fast, underwent routine physical examination, and completed a questionnaire regarding bowel habits (Irritable Bowel Syndrome (IBS) ROME III criteria and IBS Quality of Life (IBS-QOL) questionnaires)^33^. After an overnight fast, blood samples were drawn for plasma biochemistry and hematology, markers of inflammation, lipid and glucose metabolism, liver enzymes, and kidney function. Also, at baseline and after 4 weeks, resting energy expenditure (REE) as well as glucose and lipolysis fluxes were determined during a 2-step hyperinsulinemic euglycemic stable isotope-based clamp, and non-invasive magnetic resonance spectroscopy (MRS) of the liver was performed to measure IHTG content, as described in the Supplementary Notes. Finally, at baseline, 4 weeks, and 6 weeks, study participants collected and stored a morning fecal sample in their home freezer (−20° C) and brought all vials in frozen form to the study center at the end of the trial. Fecal samples were subsequently stored at −80° C for microbiota analyses.

### Power calculation and statistical analysis

Based on the effect size seen upon lean donor FMT in a previous study^5^, as well as on the variance of the clinical measurements (stable isotope hyperinsulinemic clamp), we estimated that at least eight subjects per group were needed. A sample size of nine subjects per dose group was chosen to account for potential drop-outs. Within-group changes were tested with paired Wilcoxon-signed rank tests, while Mann–Whitney *U-*tests were used to compare independent groups. Kruskal–Wallis tests were used for between-group comparisons of baseline characteristics as well for between-group comparisons of relative changes. Spearman’s rho was used for correlation analysis. The significance level (alpha) used was 0.05. Power calculations and statistics are described in further detail in Supplementary Notes.

### Fecal SCFA and plasma bile acid measurements

Fecal SCFAs were measured in fresh (directly frozen at −20° C) morning stool samples at baseline and 4 weeks. SCFAs were separated using liquid–liquid extraction and measured using high-performance liquid chromatography-ultraviolet (HPLC-UV)^34^. Plasma bile acids were measured in fasting plasma at baseline, 4 weeks, and 6 weeks after treatment. SCFA and bile acid measurements are described in detail in Supplementary Notes.

### Strain-specific qPCR

The qPCR target DNA region was a unique sequence present in *A. soehngenii* strain L2-7 (position 2157026-2155995, coding for a hypothetical protein) that was selected based on (i) its presence in the genome of *A. soehngenii* L2-7 and absence in *A. hallii* and other *Anaerobutyricum* (meta)genomes, (ii) the usefulness of primers that were obtained by Primer-Blast, including a start with 1–2 GC pairs, melting temperature between 50–60° C, not >5° C difference between the melting temperatures of the forward/reverse primers, and absence of primer complementary regions, and (iii) successful amplification in samples with *A. soehngenii* L2-7 (with appropriate melting temperature of the qPCR amplicons) and the absence of amplification in negative controls. The minimal detection level was set to 3 × 10^−5^ ng DNA (corresponding to around 7000 gene copies), and the standard curves had a dynamic range of up to 1 ng DNA. Standards of *A. soehngenii* DNA ranging from 2 × 10^−5^ ng of DNA (corresponding to around 5000 gene copies) to 8 ng of DNA (2 × 10^9^ gene copies) were used. The baseline samples taken prior to the *A. soehngenii* intervention were all below the detection level. The gene copies were determined based on the standard curve of *A. soehngenii* L2-7 DNA.

### Fecal microbiota analyses

DNA was extracted from fecal samples taken at baseline, 4 weeks, and 6 weeks after treatment. Subsequent shotgun metagenomic sequencing was performed by Clinical Microbiomics (Copenhagen, Denmark) on an Illumina HiSeq 2500 using paired-end 150 bp reads. Single-nucleotide variants (SNVs) distinguishing between the administered *A. soehngenii* strain and endogenous *Anaerobutyricum* spp. were identified. *Anaerobutyricum* spp. growth dynamics in the administered drink, as well as in subject feces were calculated using the peak-to-trough ratio method by inferring replication activity^35^. Metagenomic data was also used to estimate the ratio of secreted/ingested *A. soehngenii* cells. All DNA extraction, library preparation, and metagenomic methods are described in detail in Supplementary Notes.

### Reporting summary

Further information on research design is available in the Nature Research Reporting Summary.

## Supplementary information

Supplementary Information

Reporting Summary

Supplementary Data 1

## Data Availability

All raw sequencing data are deposited to the European Genome-phenome Archive, with study accession number E8GAS0000100349 and dataset accession number EGAD00001004849.
