# Supplementary material for: Treatment with Anaerobutyricum soehngenii: a pilot study of safety and dose–response effects on glucose metabolism in human subjects with metabolic syndrome
Source: NPJ Biofilms Microbiomes. 2020 Mar 27;6:16. doi: 10.1038/s41522-020-0127-0 (PMC7101376; doi:10.1038/s41522-020-0127-0)
Supplement: Supplementary file 1 — Supplementary Information [file 41522_2020_127_MOESM1_ESM.pdf]

Supplementary Notes

Supplementary Data Set 1. Screening and baseline measurements used as study inclusion criteria for each study subject.

Study design

Subjects were assigned to 1 of 3 treatment arms (Figure 1 in main text) where they consumed once-daily for 4 weeks 10 ml of *A. soehngenii*[1] at a concentration of either 10^7 cells/day (low dose), 10^9 cells/day (middle dose), or 10^{11} cells/day (high dose). For each dose (starting with 10^7 cells/day ingested with 100 ml milk), 1 study subject completed the full study duration of 4 weeks without occurrence of adverse events before the next 8 subjects using the same dose were included. Based on the effect size seen upon lean donor FMT in a previous study[2] as well as the variation (SD) of the clinical measurements (stable isotope hyperinsulinemic clamp) we estimated that at least 8 per group were needed. A sample size of 9 subjects per dose group was chosen so as to account for potential drop-outs. Following completion of 9 subjects in a study arm, starting with the lowest dose, the decision for the introduction of the next dosage level was taken by the investigators and the clinical pharmacist/clinical pharmacologist. These steps were repeated for the other treatment arms. Compliance was verified by counting empty vials returned during the study visit and by analyzing the administered *A. soehngenii* in the stools of the volunteers. Following informed consent and screening, subjects visited the clinical research unit at baseline, after 1, 2, and 4 weeks of treatment, and 1 and 2 weeks after completion of treatment. Subjects were instructed to record dietary habits online (mijn.voedingscentrum.nl/nl/eetmeter) the week before baseline measurements and throughout the study period.

2-step hyperinsulinemic euglycemic clamp and resting energy expenditure (REE)

Oxygen consumption and CO_2 production were measured continuously for 20 minutes using a ventilated hood system (Vmax Encore 29; SensorMedics, Anaheim, CA). REE, carbohydrate, and fatty acid oxidation rates were calculated.[3] Basal and insulin-mediated glucose fluxes were determined during a 2-step hyperinsulinemic euglycemic clamp with stable isotopes.[4] Subjects were admitted to the clinical research unit following an overnight fast. Intravenous catheters were inserted in the peripheral veins of both arms. One catheter was used to infuse the tracers [6,6-2H2]glucose and [1,1,2,3,3-^{2}H5]glycerol (99% enriched; Cambridge Isotopes, Andover, MA, USA), 20% glucose enriched with 1% [6,6-^{2}H2]glucose, and insulin (Actrapid; Novo Nordisk Farma, Alphen aan de Rijn, The Netherlands). The other catheter was used for sampling blood, which was arterialized using a heated-hand box at 57C. At 2 h before starting the clamp (t=−2 h), a primed continuous infusion of both [6,6-^{2}H2]glucose and [1,1,2,3,3-^{2}H5]glycerol was started and continued until the end of the experiment. After 2 h (t=0), infusion of insulin was started at a rate of 20 mU·m−2(body surface area)·min−1. Plasma glucose was measured every 10 min using a glucose analyzer (YSI 2300 Stat Plus Glucose Lactate Analyzer, YSI Life Sciences, Yellow Springs, Ohio). In order to keep plasma glucose at 5 mmol·l-1, 20% glucose enriched with 1% [6,6-^{2}H2]glucose was infused at a variable rate. Insulin infusion was increased after 2 h of insulin infusion (t=2 h) to 60 mU·m-2·min-1. At t=0, 2 h, and 4 h, 5 blood samples were taken to assess glucose, glycerol enrichments and free fatty acids (FFA). Rates of appearance (Ra) and Rates of disposal (Rd) of glucose and glycerol were calculated using the modified forms of the Steele equations for (non-)steady state measurements.[4]

Liver Magnetic Resonance Spectroscopy

Intrahepatic triglyceride (IHTG) content was measured by using Magnetic Resonance Spectroscopy (MRS) performed on a clinical 3.0 T Philips Intera scanner (Philips Healthcare, Best, The Netherlands). First, T1-weighted coronal and axial localizer images of the abdomen were obtained which were then used to position a voxel of 20 x 20 x 20 mm. Because the diaphragm, edges of the liver or other vascular and biliary structures must be avoided, the voxel was usually placed in the right hepatic lobe. For all subjects, time of acquisition and voxel size were standardized. Spectra were obtained by using first-order iterative shimming, a PRESS sequence with relaxation time/echo time (TR/TE) = 35/2000 ms and 64 signal acquisitions during free breathing. The liver H-MR spectra were evaluated by using jMRUI software. To quantify the lipid signal resonances, water non-suppressed spectra were used. Relative fat content was expressed as a ratio of the fat peak area over the cumulative water and fat peak areas (1.3 ppm / (1.3 ppm + 4.65 ppm)). Calculated peak areas of water and fat were corrected for T2 relaxation.[5,6,7]

Fecal SCFA and plasma bile acid measurements

Plasma bile acid concentrations were determined using LC-MS/MS system.[8] Fecal short-chain fatty acids (SCFA) (butyrate, propionate, and acetate) were separated using liquid-liquid extraction and measured using HPLC-UV in fecal samples taken before and after 4 weeks of treatment.[9] Samples were vortexed and equilibrated at room temperature for 5 min before the addition of 100 ul of concentrated HCl, followed by vortexing to 15 s. The extraction time was 20 min in 5 ml diethylether. After 5 min centrifugation at 200 g the supernatant was transferred to a different pyrex extraction tube and 500 ul of NaOH 1M was added. Samples were then again extracted for 20 min, then centrifuged. The resulting aqueous phase was transferred to an autosampler vial and mixed with 100 uL concentrated HCl before injection to the HPLC-UV. [6,6-^{2}H2] glucose enrichment was measured.[10] To calculate the[6,6-2H2]glucose enrichment in the samples, the glucose pentaacetate derivative was also prepared and the M2 was measured. The glucose pentaacetate derivative was used instead of the aldonitrile pentaacetate derivative, because for this derivative it is easier to keep the values of unenriched glucose within the 3% levels of the theoretical ones, and this derivative gives two peaks for glucose (α- and β-anomer), resulting in a duplicate value for one sample. The fractional GNG was calculated as: fractional GNG = EM1/A11, where A11 is the maximum EM1 that can be reached for the measured precursor pool enrichment. [1,1,2,3,3-^{2}H5]glycerol was also measured.[11]

Intestinal microbiota analyses

Sample DNA extraction and library preparation

DNA extraction from fecal samples for shotgun metagenomics was performed as follows.[2,12] Samples were thawed on ice and homogenized before weighting of aliquots prior to DNA extraction. The incubation temperature after the bead beating was raised from 70C to 95C to further enhance the cell breakage. The amount of starting fecal mass was 250 mg. In the homogenization step we added a few 3 mm glass beads to prevent the sedimentation of the 0.1 mm beads and the fecal solids in order to assure uniform sample processing. Repeated bead beating was performed using a FastPrep®-24 instrument at 5.5 ms^{-1} with a CryoPrep™ adapter (MP Biomedicals, Inc., USA) and 0.1 mm zirconium–silica beads (Biospec Products, Bartlesville, OK, USA). The beat beating treatment used had a duration of 6 min (2 x 3 min). DNA purification was performed using a Promega Maxwell 16 Tissue LEV Total RNA Purification Kit, Custom (Promega, Madison, U.S.), optimized for gDNA extraction from fecal samples by excluding the DNase step according to Promega recommendations for optimal extraction of DNA from fecal samples. Elution of DNA from the purification columns was done twice to improve the DNA yield. Elution of DNA from the purification columns was done twice to improve the DNA yield. DNA was eluted in 50 ul of nuclease free water and quantified by Nanodrop (ThermoScientific) Subsequent shotgun metagenomic sequencing was done by Clinical

Microbiomics, Copenhagen, Denmark. Before sequencing, the quality of the DNA samples was evaluated using agarose gel electrophoresis, NanoDrop 2000 spectrophotometry and Qubit 2.0 fluorometer quantitation. The genomic DNA was randomly sheared into fragments of around 350 bp. The fragmented DNA was used for library construction using NEBNext Ultra Library Prep Kit for Illumina (New England Biolabs). The prepared DNA libraries were evaluated using Qubit 2.0 fluorometer quantitation and Agilent 2100 Bioanalyzer for the fragment size distribution. Quantitative real-time PCR (qPCR) was used to determine the concentration of the final library before sequencing. In addition, qPCR was performed to quantify the total *A. soehngenii* levels (primers L2-7_20-5f 5';-ATGCCAGACGAGGATGAAGG-3'; and L2-7 20-r 5';-TCTCCTTCCGGCTTTCCTGT-3';) in the fecal samples taken at baseline, 4 weeks, and 6 weeks after treatment.

Sample DNA sequencing and quality control

The library was sequenced using 2 x 150 bp paired-end sequencing on an Illumina HiSeq HiSeq2500 platform. Raw reads were quality controlled using KneadData 0.5.4 together with Trimmomatic. The reads were quality trimmed by removing the first 10 bp, cutting reads at the 3'-end with a sliding window of 4 bp. Only high quality (HQ) reads with a minimum length of 60 bp and with a mean Phred score of 15 or better were used for mapping. Furthermore, sequence reads that mapped to the human reference genome (using Bowtie2) were discarded. Thereafter, all samples were downsized to 20 million reads to ensure comparable sensitivity across samples.

Profiling of metagenomics species

The Clinical Microbiomics database of 1,507 adult human gut metagenomic species (MGSs) was used as reference for profiling the composition of the samples. These 1,507 MGSs were previously identified based on the co-abundance of genes across 2,300 human stool samples, using the non-redundant Integrated Catalogue of Reference Genes (IGC) for the human gut microbiome as a reference.[13] On average, 76% of all high-quality (base call accuracy > 99.9%) non-human reads mapped to the IGC; however, around 20% of the mapped reads mapped ambiguously (i.e. to multiple genes). To improve profiling precision, these ambiguous reads were removed and considered unmapped reads. Thus the proportion of mapped reads per samples was between 60-65%. Clusters of genes that passed quality assessment (> 700 genes, inter-gene Pearson correlation coefficient > 0.9, GC content interquartile range < 10%, observed in ≥ 5 samples) were defined as metagenomic species (MGSs). To calculate relative abundances of the MGSs, we mapped the HQ nonhuman sequencing reads to the IGC, requiring 95% identify and 90% coverage. Ambiguously mapped reads were removed. Based on these mappings, we calculated the length-normalized gene depths (reads/bp) for all IGC genes. Then, MGS depths were calculated as the average gene depths across each MGS gene set. The MGS depths were then scaled relative to the total number of HQ non-host sequences reads, including the reads that did not map to IGC (scaling factor = all MGS mapped reads / all reads). Furthermore, an MGS was considered detected only if HQ reads were mapped to at least 3 of its 100 core genes, defined as genes that proved particularly robust for abundance profiling throughout a 2300 sample reference. MGSs that did not satisfy this criterion were set to zero abundance.

Differentiating the administered *A. soehngenii* from the endogenous *Anaerobutyricum* spp. using SNVs

The reference genome of the administered *A. soehngenii*[14] was almost identical to the endogenous *A. hallii* present in the subjects before the intervention. In order to estimate the relative abundance of the administered *A. soehngenii* in the post-treatment samples, Single Nucleotide Variants (SNVs) distinguishing between the administered strain and the endogenous strains were identified.[15] For each subject, a unique subset of discriminatory SNVs was used to discriminate between the administered and endogenous strains using the

proprietary method of Clinical Microbiomics A/S described below. The discriminatory subset of SNVs was defined as all SNVs present in the administered strain but never present in the endogenous strains (before-treatment sample), or the other way around. SNV calling was done using SAMtools and BCFtools. SNVs were filtered, requiring that a SNV was observed with a minimum depth of 5 in 1 sample with an allele frequency (allele depth / total depth) of at least 0.8. We then used the proportion of reads mapping to these SNVs to estimate the abundance of each strain in the post-treatment sample. The discriminatory SNV analysis requires that the endogenous *Anaerobutyricum* spp. are sufficiently abundant in the pre-treatment specimen to produce sufficient reads to call discriminative SNVs.

Replication activity of *Anaerobutyricum* spp.

Anaerobutyricum spp. growth dynamics were calculated using peak-to-trough ratio.[16] Replication activity was assessed in each sample by mapping the reads to the *A. soehngenii* reference genome[14] and assessing the depth of sequencing along the genome as a surrogate for local replication status. Replication signal is defined as the ratio of the sequencing depth at the origin of replication to that at the terminus. Therefore, the replication signal minus 1 can be interpreted as an estimate of the proportion of cells undergoing replication. It was not possible to discriminate between the endogenous *Anaerobutyricum* spp. and the administered *A. soehngenii* with regard to replication signal. Replication activity of *A. soehngenii* in the administered drink was determined after freezing, storage, and thawing, in order to estimate the replication activity at the moment of administration.

Power calculation and statistics

Since this was the first pilot study aiming to assess safety and effect on insulin sensitivity of each administered *A. soehngenii* dose in human insulin resistant subjects, a formal power calculation was not possible but we based the potential effect size on our previous study.[2] Moreover, based on our findings in insulin-resistant mice treated for 28 days with increasing concentrations of *A. soehngenii*[17], we hypothesized that 8 metabolic syndrome subjects per group were needed to detect a 30% significant step-wise increase in peripheral insulin sensitivity (as assessed by hyperinsulinemic clamp.[2] Taking a 10% dropout range into account, it was thus estimated that a total of 27 (3 x 9) subjects were needed (with 0.05 2-sided significance levels with 90% power). As a normal distribution could not be assumed, all data is presented as median [interquartile range]. Within-group changes between baseline and 4 weeks were tested with paired Wilcoxon signed-rank tests, while Mann-Whitney U tests were used to compare independent groups. Kruskal-Wallis tests were used for between-group comparisons of baseline characteristics as well for between-group comparisons of relative changes from baseline to week 4. Spearman’s rho rank correlation was used for (non-parametric) correlation analysis. The significance level (alpha) used in the analysis was 0.05.

Calculation of the ingested / secreted *A. soehngenii*

The ratio of secreted / ingested *A. soehngenii* cells was calculated by estimating 10^{11} total bacterial cells/g feces and 100 g feces produced per day.[18] The absolute numbers of *A. soehngenii* cells excrete daily in feces was calculated by multiplying the estimated mass of feces produced per day by the estimated bacterial cell density per g of feces and by the relative abundance of *A. soehngenii* inferred from shotgun sequencing and qPCR measurements. The absolute numbers of *A. soehngenii* ingested daily was calculated by multiplying the know concentration of *A. soehngenii* cells in the administered drink by the daily volume of ingested drink.

References

1. Shetty, S. A. et al. Reclassification of *Eubacterium hallii* as *Anaerobutyricum hallii* gen. nov., comb. nov., and description of *Anaerobutyricum soehngenii* sp. nov., a butyrate and propionate-producing bacterium from infant faeces. *Int J Syst Evol Microbiol* **68**, 3741–3746.(2018).
2. Vrieze, A. et al. Transfer of intestinal microbiota from lean donors increases insulin sensitivity in individuals with metabolic syndrome. *Gastroenterology* **143**, 913-916 (2012).
3. Frayn, K. N. Calculation of substrate oxidation rates in vivo from gaseous exchange. *J Appl Physiol* **55**, 628–634 (1983).
4. Steele, R. Influences of glucose loading and of injected insulin on hepatic glucose output. *Ann N Y Acad Sci* **82**, 420–430 (1959).
5. Van Werven, J. R. et al. Reproducibility of 3.0 Tesla magnetic resonance spectroscopy for measuring hepatic fat content. *J Magn Reson Imaging* **30**, 444–448 (2009).
6. Bouter, K. E. et al. Differential metabolic effects of oral butyrate treatment in lean versus metabolic syndrome subjects. *Clin Transl Gastroenterol* **9**, e155 (2018).
7. Koopman, K. E. et al. Hypercaloric diets with increased meal frequency, but not meal size, increase intrahepatic triglycerides: A randomized controlled trial. *Hepatology* **60**, 545–553 (2014).
8. Vrieze, A. et al. Impact of oral vancomycin on gut microbiota, bile acid metabolism, and insulin sensitivity. *J Hepatol* **60**, 824-831 (2013).
9. De Baere, S. et al. Development of a HPLC–UV method for the quantitative determination of four short-chain fatty acids and lactic acid produced by intestinal bacteria during in vitro fermentation. *J Pharm Biomed Anal* **80**, 107–115 (2013).
10. Ackermans, M. T. et al. The quantification of gluconeogenesis in healthy men by $^{2}H_2O$ and[2-^{13}C]glycerol yields different results: rates of gluconeogenesis in healthy men measured with $^{2}H_2O$ are higher than those measured with [2-^{13}C]glycerol[1]. *J Clin Endocrinol Metab* **86**, 2220–2226 (2001).
11. Patterson, B. W. et al. Use of stable isotopically labeled tracers to measure very low density lipoprotein-triglyceride turnover. *J Lipid Res* **43**, 223–233 (2002).
12. Salonen, N. J. et al. Comparative analysis of fecal DNA extraction methods with phylogenetic microarray: Effective recovery of bacterial and archaeal DNA using mechanical cell lysis. *Microbiol Methods* **81**, 127-134 (2010).
13. Li, J. et al. An integrated catalog of reference genes in the human gut microbiome. *Nat Biotechnol* **32**, 834–841 (2014).
14. Shetty, S. A. et al. Complete genome sequence of *Eubacterium hallii* strain L2-7. *Genome Announc* **5**, e01167-17 (2017).
15. Li, S. S. et al. Durable coexistence of donor and recipient strains after fecal microbiota transplantation. *Science* **352**, 586–9 (2016).
16. Korem, T. et al. Growth dynamics of gut microbiota in health and disease inferred from single metagenomic samples. *Science* **349**, 1101–1106 (2015).
17. Udayappan, S. *et al.* Oral treatment with *Eubacterium hallii* improves insulin sensitivity in db/db mice. *npj Biofilms Microbi* **2**, 16009 (2016).
18. Sender, R., Fuchs, S., & Milo, R. Revised estimates for the number of human and bacteria cells in the body. *PLoS Biol* **14**, e1002533 (2016).

Supplementary Figures

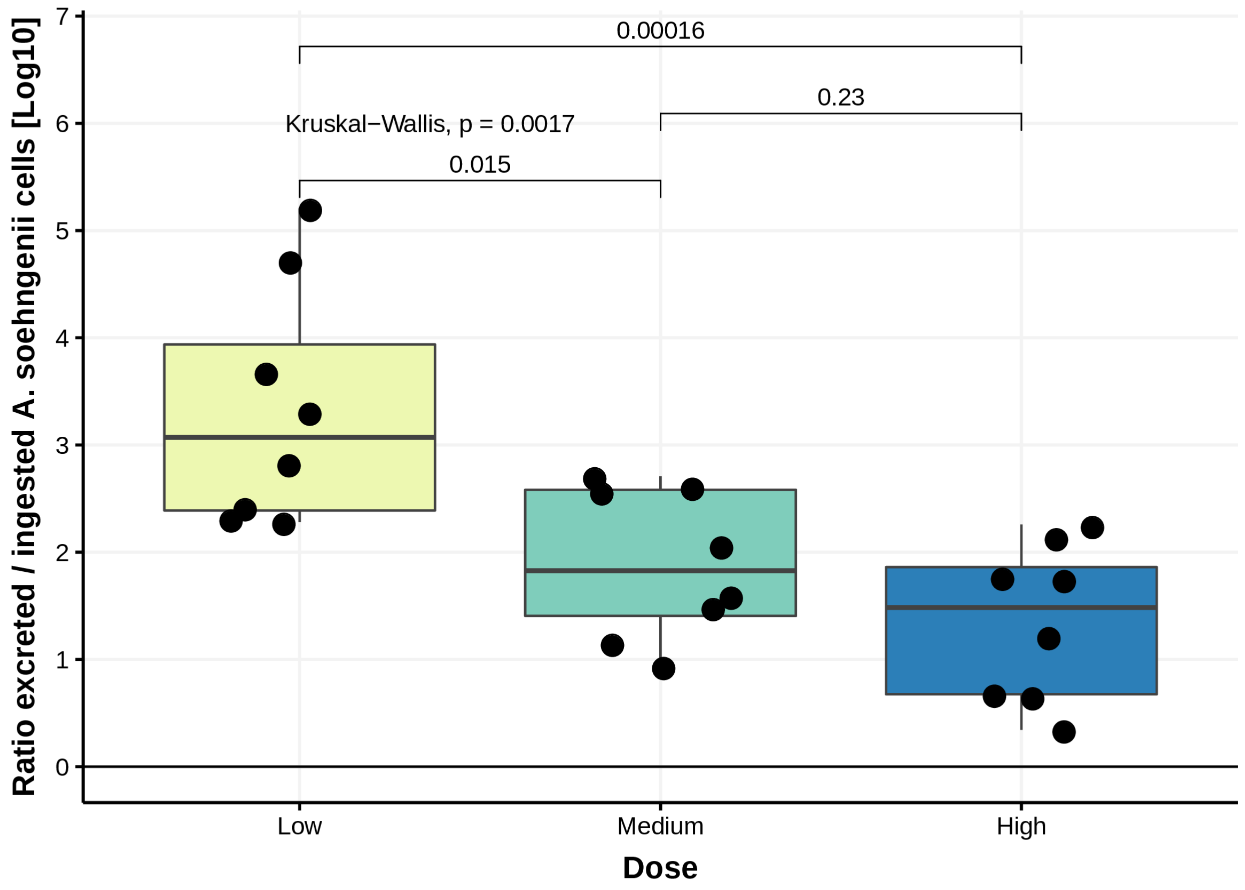

Supplementary Fig. 1. Ratio of excreted to ingested *A. soehngenii* per dose group

The ratio of secreted / ingested A. soehngenii cells was calculated by estimating 10^{11} total bacterial cells/g feces and 100 g feces produced per day. Data expressed as median and [interquartile range]. P-values shown for between-group comparisons (Mann-Whitney U tests) and in between-group comparison (Kruskal-Wallis test).

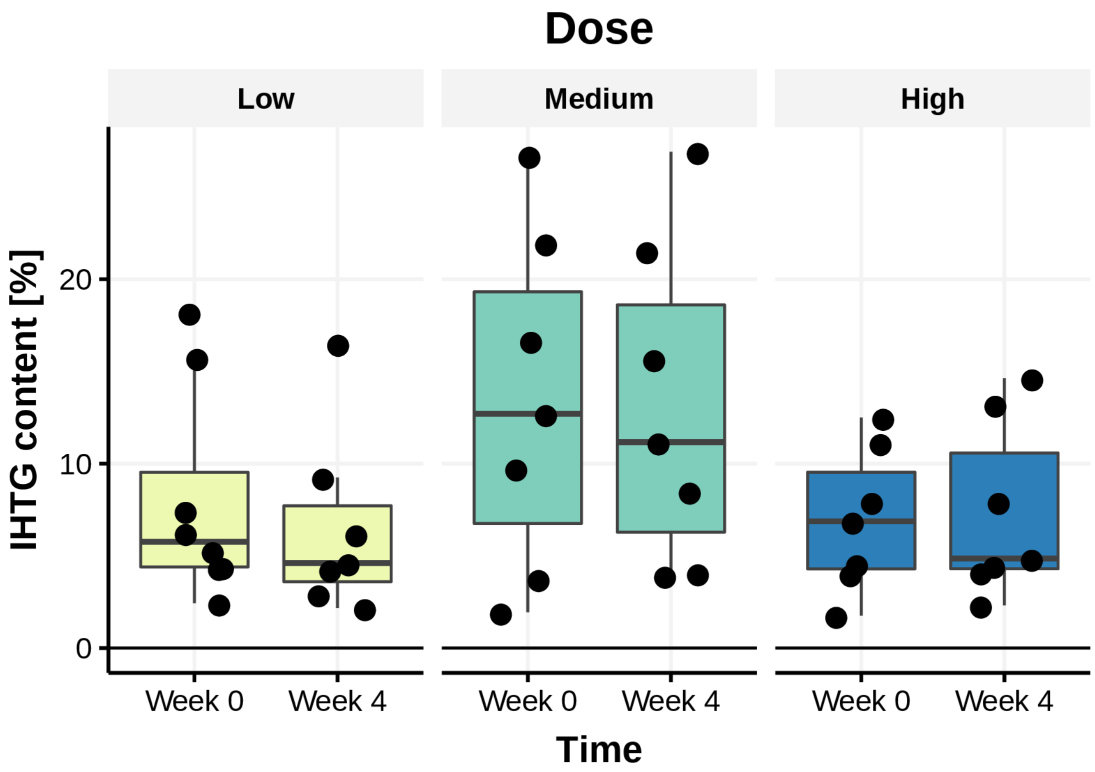

Supplementary Fig. 2. Intrahepatic triglyceride content (IHTG) after 4 weeks of treatment, shown per dose group
Data expressed as median [interquartile range]. Abbreviations: IHTG = intrahepatic triglyceride

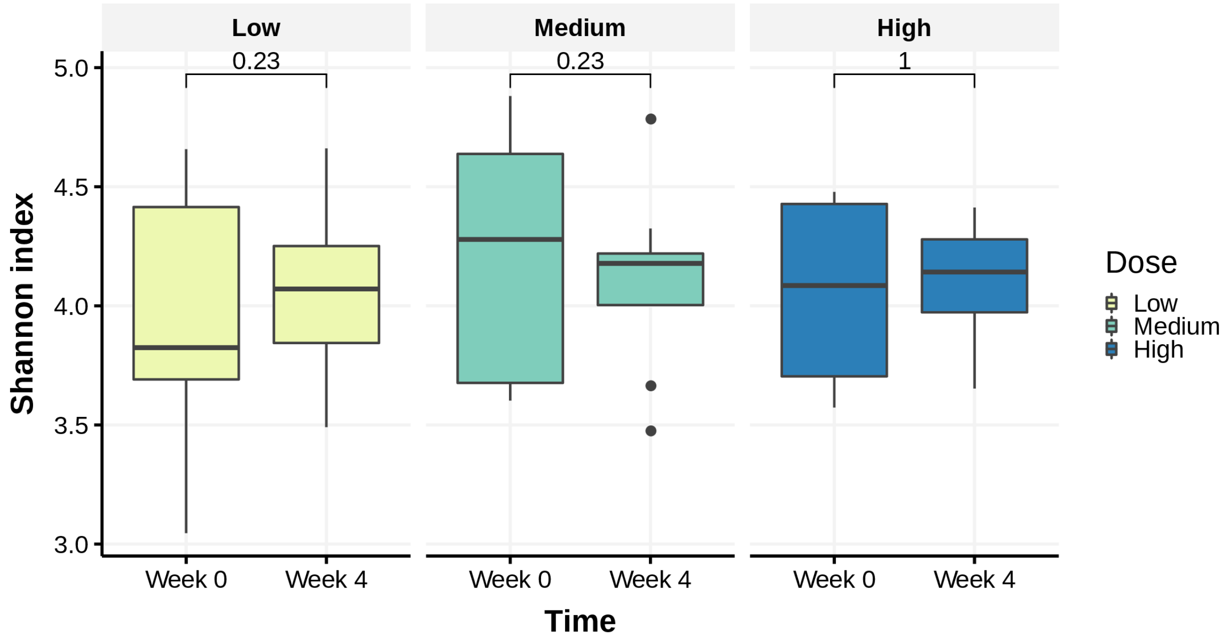

Supplementary Fig. 3. Microbiota alpha diversity

Fecal microbiota alpha-diversity measured using the Shannon index at week 0 (baseline) and 4 weeks upon low, middle, and high dose of A. soehngenii treatment.

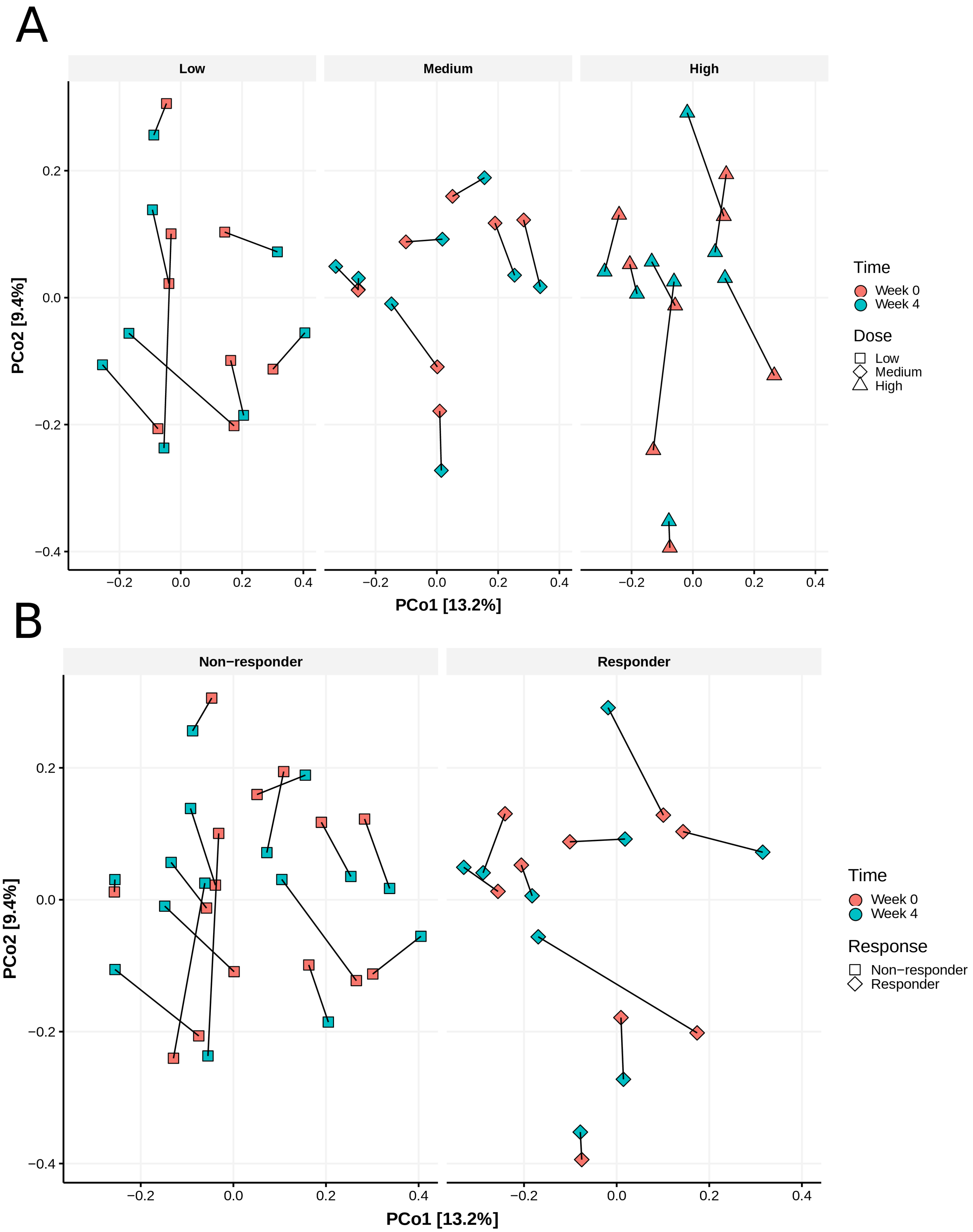

Supplementary Fig. 4. Principal Component Analysis (PCoA) based on Bray-Curtis distances calculated from fecal microbial composition. A) faceted by dose group; and B) faceted by response status.

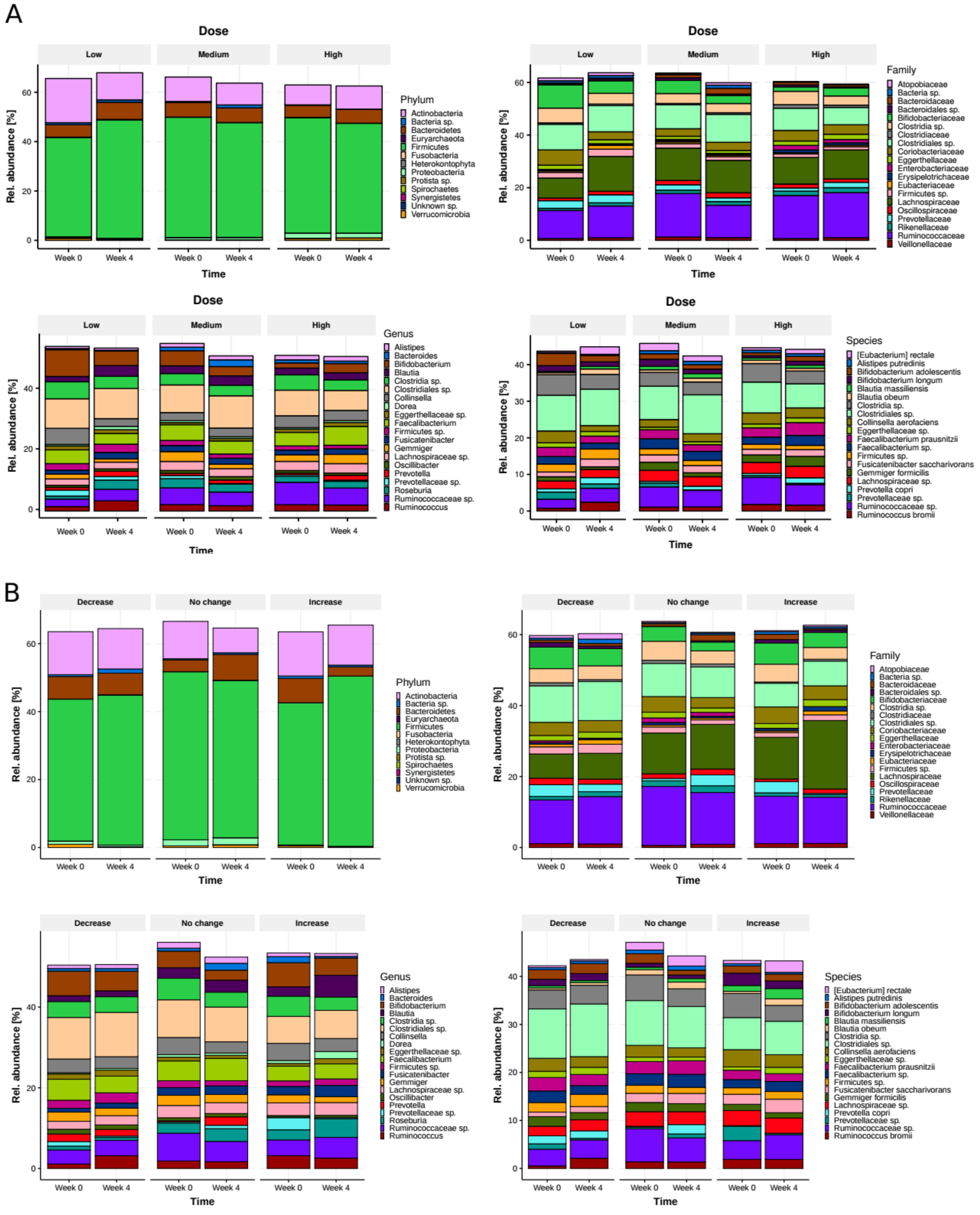

Supplementary Fig. 5. Microbiota composition profiles at baseline and after 4 weeks of treatment, stratified A) per dose group; and B) by change in Rd. Subjects whose Rd increased by at least 4 μmol/kg/min were classified as showing an "Increase" in Rd; subjects whose Rd decreased by at least 4 μmol/kg/min were classified as showing a "Decrease"; and subjects whose Rd changed by less than 4 μmol/kg/min (either increasing or decreasing) were labeled as showing "No change".

All phyla and the 20 most abundance families, genera, and species, respectively, shown as % relative abundance of the total fecal microbiome.

Supplementary Tables

Supplementary Table 1. Side effects and questionnaires on bowel habits at baseline, 4 weeks and 6 weeks
Reported side effects and questionnaires regarding bowel habits: IBS ROME III diagnostic criteria (minimum score 0 = does not fulfill criteria of IBS) and IBS-QOL (34 items, score from 1 = not at all, to 5 = a great deal, minimum score 34 = maximum quality of life).

	**10^7 cells/day**	**n = 8**		**10^9 cells/day**	**n = 8**		**10^{11} cells/day**	**n = 8**	
	Week 0	Week 4	Week 6	Week 0	Week 4	Week 6	Week 0	Week 4	Week 6
IBS ROME III criteria	0	0	0	0	0	0	0	0	0
IBS-QOL	34	34	34	34	34	34	34	34	34
Side effects									
- Nausea	0	0	0	0	0	0	0	0	0
- Flatulence	0	0	0	0	0	0	0	0	0
- Cramps	0	0	0	0	0	0	0	0	0
- Borborygmi	0	0	0	0	0	0	0	0	0
- Gastric reflux	0	0	0	0	0	0	0	0	0

Supplementary Table 2. Fecal *A. soehngenii* levels (qPCR) at baseline, 4 weeks, and 6 weeks. Data expressed as median [interquartile range].

	**10^7 cells/day** Week 0	**n = 8** Week 4	Week 6	**10^9 cells/day** Week 0	**n = 8** Week 4	Week 6	**10^{11} cells/day** Week 0	**n = 8** Week 4	Week 6
qPCR fecal *A. soehngenii*, *ng/µl x10^{-5}*	0.8 [0.4-1.7]	6.1 [2.2-11.2]	0.8 [0.4-1.8]	0.4 [0.1-1.0]	747.8 [351.2-1378.2]	0.9 [0.6-2.7]	0.6 [0.2-1.7]	8364.3 [3177.4-16592.9]	0.7 [0.1-3.1]
Gene copies *A. soehngenii* / g feces [log10]	6.26 [6.12-6.57]	7.12 [6.73-7.52]	6.27 [6.06-6.61]	5.92 [5.36-6.35]	9.23 [8.94-9.42]	6.28 [6.08-6.77]	6.15 [5.69-6.53]	10.30 [9.96-10.60]	6.18 [5.39-6.60]

Supplementary Table 3. Circulating insulin levels during clamp tests at week 0 and week 4
Data expressed as median and [interquartile range]. P-values between the 3 groups and between week 0 and 4 did not reach significance ($p < 0.05$).

	Basal state			Step 1			Step 2		
	10^7 cells/day	10^9 cells/day	10^{11} cells/day	10^7 cells/day	10^9 cells/day	10^{11} cells/day	10^7 cells/day	10^9 cells/day	10^{11} cells/day
**Clamp week 0**									
Insulin, *pmol/l*	86 [60-139]	98 [78-168]	79 [40-186]	359 [293-394]	352 [313-456]	353 [222-422]	891 [744-1018]	917 [675-1005]	827 [599-967]
**Clamp week 4**									
Insulin, *pmol/l*	79 [52-141]	126 [71-163]	102 [47-132]	335 [239-432]	375 [284-487]	356 [220-432]	819 [640-1077]	964 [675-1116]	841 [595-956]

Supplementary Table 4. Glucose metabolism and other metabolic parameters

Data expressed as median [interquartile range]. p*: p-value of change between the three dose groups; p^: percentage change between the three dose groups.

Abbreviations: EGP = endogenous glucose production; Rd = rate of disappearance; Ra = rate of appearance; FFA = free fatty acid; REE = resting energy expenditure; IHTG = intrahepatic triglyceride

	**All subjects** Week 0	**n = 24** Week 4	**p**	**10^7 cells/day** Week 0	**n = 8** Week 4	**p**	**10^9 cells/day** Week 0	**n = 8** Week 4	**p**	**10^{11} cells/day** Week 0	**n = 8** Week 4	**p**	**p***	**p^**
Glucose Rd, *µmol/kg/min*	33.0 [27.5-43.5]	32.2 [28.1-41.4]	0.303	36.7 [28.2-48.7]	36.7 [26.4-48.5]	0.161	30.2 [21.2-35.0]	30.1 [27.2-33.5]	0.779	34.8 [26.9-47.1]	35.5 [29.7-45.0]	1.00	0.50	0.32
EGP (fasting), *µmol/kg/min*	8.3 [7.9 – 9.1]	8.3 [7.7-9.1]	0.796	8.6 [7.9-9.5]	8.6 [8.0-9.5]	0.779	8.8 [7.6-9.1]	8.2 [7.5-9.4]	0.612	8.2 [7.6-9.3]	8.3 [7.5-9.1]	0.48	0.48	0.41
EGP suppression, *%*	74.4 [68.4-82.6]	77.2 [69.7-85.7]	0.345	69.7 [64.7-79.4]	79 [65.1-87.6]	0.484	74.4 [70.0-86.4]	76.9 [69.3-86.2]	0.401	81.6 [67.8-85.9]	75.8 [72.4-86.0]	0.88	0.88	0.98
Glycerol Ra suppression, *%*	65.1 [54.8-72.3]	69.1 [56.7-77.3]	0.493	58.4 [51.7-77.6]	72.5 [57.5-78.5]	0.401	66.9 [57.8-77.6]	61.3 [48.6-71.3]	0.161	67.1 [52.4-70.6]	74.9 [54.3-80.5]	0.16	0.14	0.17
FFA suppression, *%*	83.2 [73.0-85.7]	81.0 [76.4-86.4]	0.422	83.3 [80.5-86.9]	83.4 [78.0-86.7	0.484	77.8 [71.8-85.5]	76.8 [69.3-82.8]	0.575	83.6 [71.8-90.2]	83.7 [76.6-90.3]	1.00	0.78	0.86
REE, *kcal/day*	1730 [1652-1987]	1774 [1705 -1925]	0.945	1704 [1600-2053]	1774 [1762-1971]	0.866	1864 [1719-2074]	1853 [1657-1981]	0.753	1679 [1635-1939]	1765 [1667-1935]	0.48	0.84	0.84
IHTG, *%*	7.2 [4.4-12.7]	6.2 [4.1-13.2]	0.734	5.8 [4.4-13.7]	4.6 [2.9-9.3]	0.866	12.7 [3.8-22.0]	11.2 [4.1-21.5]	1.000	6.9 [4.0-11.1]	4.9 [4.1-13.2]	0.31	0.71	0.55

Supplementary Table 5. Correlations between clinical markers of insulin sensitivity and administered *A. soehngenii*

Correlations between clinical markers of insulin sensitivity and the endogenous and administered *A. soehngenii* are shown. Spearman's rho correlation and corresponding p-values are shown. *Abbreviations: EGP = endogenous glucose production; Rd = rate of glucose disappearance; REE = resting energy expenditure*

Clinical marker	rho	p
**Rd**	**0.41**	**0.044***
**Rd change**	**0.39**	**0.061**
**Relative Rd change**	**0.40**	**0.054**
EGP suppression	0.123	0.568
EGP suppression change	-0.137	0.525
Relative EGP suppression change	-0.062	0.774
REE change	-0.017	0.942
Relative REE change	-0.009	0.969

Supplementary Table 6. Fecal SCFA levels before and after *A. soehngenii* treatment in all dosage groups

Data are expressed as median [interquartile range]. P-values represent within group changes between week 0 and 4 (p, analyzed using a Wilcoxon test). A p-value < 0.05 was considered significant. *Abbreviations: SCFA = short chain fatty acid*

	**All subjects** Week 0	**n = 24** Week 4	**p**	**10^7 cells/day** Week 0	**n = 8** Week 4	**p**	**10^9 cells/day** Week 0	**n = 8** Week 4	**p**	**10^{11} cells/day** Week 0	**n = 8** Week 4	**p**
Fecal SCFA												
-Acetic acid, *umol/g*	404 [318-548]	420 [306-532]	0.846	356 [307-481]	360 [196-626]	0.735	372 [288-636]	328 [255-457]	0.310	523 [420-663]	508 [339-734]	0.401
-Butyric acid, *umol/g*	84 [54-151]	79 [47-122]	0.370	62 [46-88]	74 [23-168]	0.612	64 [7-156]	60 [42-112]	0.398	132 [87-161]	114 [52-150]	0.263
-Propionic acid, *umol/g*	176 [144-226]	162 [124-200]	0.337	157 [137-179]	99 [39-324]	0.499	178 [137-264]	161 [122-195]	**0.028**	201 [146-243]	199 [142-215]	0.401

Supplementary Table 7. Plasma bile acids after 4 and 6 weeks

Shown are bile acids measurements at week 0, 4 and 6, expressed as median [interquartile range] for all three treatment groups. P-values represent within group changes between week 0, week 4 and week 6 (p, Wilcoxon test), and change (p*) and percentage change (p^) in between the 3 groups (Kruskal-Wallis tests). A p-value < 0.05 was considered significant.

	**All subjects Week 0**	**n = 24 Week 4**	**p**	**10^7 cells/day Week 0**	**n = 8 Week 4**	**p**	**10^9 cells/day Week 0**	**n = 8 Week 4**	**p**	**10^{11} cells/day Week 0**	**n = 8 Week 4**	**p**	**p***	**p^**
Plasma total bile acids, µM	1.37 [0.85-2.18]	1.34 [1.07-2.00]	0.684	1.58 [0.82-2.53]	1.31 [1.06-2.90]	0.889	1.48 [1.15-2.10]	1.36 [1.00-2.84]	0.575	0.85 [0.62-3.05]	1.34 [0.98-2.08]	0.889	0.935	0.949
Primary bile acids, µM	0.64 [0.48-1.24]	0.71 [0.57-1.36]	0.768	0.72 [0.38-1.24]	0.736 [0.481-1.38]	0.889	0.84 [0.53-1.33]	0.64 [0.60-1.92]	0.484	0.52 [0.31-2.07]	0.78 [0.44-1.37]	0.889	0.833	0.932
Secondary bile acids, µM	0.51 [0.35-0.85]	0.58 [0.42-0.89]	0.439	0.48 [0.30-1.20]	0.661 [0.453-1.22]	0.263	0.53 [0.40-0.88]	0.59 [0.35-1.01]	0.575	0.34 [0.23-0.82]	0.53 [0.32-0.71]	1.000	0.817	0.867
Conjugated bile acids, µM	0.70 [0.47-1.34]	0.86 [0.61-1.07]	0.790	0.93 [0.45-1.96]	0.942 [0.521-1.36]	0.674	0.70 [0.47-1.42]	0.86 [0.60-1.85]	0.161	0.62 [0.40-2.01]	0.77 [0.62-0.97]	0.779	0.523	0.547
Unconjugated bile acids, µM	0.40 [0.22-0.70]	0.55 [0.37-0.72]	0.16	0.32 [0.20-0.50]	0.55 [0.34-0.73]	**0.025**	0.66 [0.34-1.05]	0.54 [0.32-0.96]	0.779	0.22 [0.17-0.94]	0.54 [0.27-1.00]	0.575	0.330	0.196
		**Week 6**			**Week 6**			**Week 6**			**Week 6**			
Plasma total bile acids, µM		2.02 [1.29-2.71]	**0.008**		1.60 [1.08-1.96]	0.889		2.55 [2.07-10.64]	**0.018**		2.06 [0.78-3.17]	0.093	**0.027**	0.061
Primary bile acids, µM		1.08 [0.46-2.07]	0.056		0.45 [0.39-1.59]	0.674		1.50 [1.14-7.83]	**0.018**		0.73 [0.47-2.09]	0.401	**0.037**	0.065
Secondary bile acids, µM		0.65 [0.56-1.26]	**0.0002**		0.61 [0.48-1.06]	0.327		1.17 [0.55-2.74]	**0.018**		0.67 [0.32-1.40]	**0.025**	**0.050**	0.149
Conjugated bile acids, µM		0.91 [0.64-1.79]	0.170		0.72 [0.51-1.06]	0.123		1.65 [0.76-2.11]	**0.018**		0.84 [0.50-2.24]	0.327	**0.007**	**0.019**
Unconjugated bile acids, µM		0.66 [0.47-1.17]	**0.0004**		0.58 [0.41-0.99]	0.091		0.91 [0.65-5.20]	**0.018**		0.48 [0.25-1.33]	**0.025**	0.358	0.449

Supplementary Table 8. Changes in the abundance of the 20 most abundant bacterial species, stratified per dose group.

Changes are shown as median [IQR] of % relative change at week 4 compared to baseline.

**Microbial species**	**Low dose [10^7 cells/day]**	**Medium dose [10^9 cells/day]**	**High dose [10^{11} cells/day]**
Alistipes putredinis	+58 [-50;+161]	-28 [-66;+15]	-12 [-51;+140]
Bifidobacterium adolescentis	-59 [-78;-24]	-16 [-67;+54]	-40 [-68;+280]
Bifidobacterium longum	-46 [-60;+17]	+18 [-40;+118]	+10 [-19;+102]
Blautia massiliensis	-10 [-82;+159]	-30 [-48;+26]	+16 [-19;+84]
Blautia obeum	+44 [-59;+140]	-4 [-67;+96]	+35 [-27;+84]
Clostridia spp.	-27 [-33;+119]	-12 [-30;+23]	-24 [-50;+32]
Clostridiales spp.	-5 [-42;+76]	+2 [-5;+30]	-11 [-35; +19]
Collinsella aerofaciens	-45 [-74;-2]	+7 [-36;+45]	-25 [-40;+53]
Eggerthellaceae spp.	+22 [-41;+144]	+12 [-40;+48]	+23 [-37;+94]
Eubacterium rectale	+340 [-84;+1312]	-13 [-64;+71]	+135 [+28;+191]
Faecalibacterium prausnitzii	-7 [-31;+66]	-41 [-65;-23]	+2 [-28;+124]
Faecalibacterium spp.	+7 [-26;+84]	-26 [-48;+46]	+50 [-13;+213]
Firmicutes spp.	+27 [-43;+120]	-5 [-23;+12]	-6 [-58;+34]
Fusicatenibacter saccharivorans	+26 [-40;+72]	-11 [-34;+15]	+2 [-14;+113]
Gemmiger formicilis	+15 [-67;+101]	-43 [-64;-36]	+43 [+14;+95]
Lachnospiraceae spp.	+3 [-70;+81]	-33 [-60;+65]	-12 [-41;+76]
Prevotella copri	-52 [-89;+16]	-47 [-96;+1112]	-33 [-90;+741]
Prevotellaceae spp.	-47 [-69;-5]	-76 [-91;+853]	+489 [-68;+2234]
Ruminococcaceae spp.	+41 [+9;+168]	-18 [-53;+7]	-11 [-42;+48]
Ruminococcus bromii	+69 [-67;+435]	-49 [-74;-3]	-27 [-61;+85]

Supplementary Table 9. Relative differences between Responders (subjects showing a significant increase in Rd) and Non-Responders (subjects showing either a significant decrease or no change in Rd) [% difference] in the baseline abundance of the 20 most abundant bacterial species.

The threshold for minimum significant change in Rd was set to 4 µmol/kg/min. In effect, subjects whose Rd increased by at least 4 µmol/kg/min were classified as showing an "Increase"; subjects whose Rd decreased by at least 4 µmol/kg/min were classified as showing a "Decrease"; and subjects whose Rd changed by less than 4 µmol/kg/min (either increasing or decreasing) were labeled as showing "No change".

Values are (median abundance $_{\text{Responder}}$ – median abundance $_{\text{Non-Responder}}$)/median abundance $_{\text{Non-responder}}$

Microbial species	Difference in Responders (sig. increase in Rd) compared to Non-Responders ([%]
Ruminococcaceae spp.	-69.7
Prevotella copri	-65.0
Clostridiales spp.	-45.6
Gemmiger formicilis	-43.0
Bifidobacterium longum	-37.7
Prevotellaceae spp.	-35.9
Eggerthellaceae spp.	-34.8
Clostridia spp.	-33.9
Faecalibacterium prausnitzii	-32.8
Firmicutes spp.	-12.2
Faecalibacterium spp.	-1.0
Lachnospiraceae spp.	+12.9
Fusicatenibacter saccharivorans	+17.5
Alistipes putredinis	+18.3
Blautia obeum	+51.4
[Eubacterium] rectale	+61.3
Collinsella aerofaciens	+74.3
Blautia massiliensis	+76.9
Bifidobacterium adolescentis	+100.0
Ruminococcus bromii	+367.8
